# A Spatiotemporally Controlled Nanoplatform for Photothermal BRD4 Degradation Enables Synergistic Cancer Immunotherapy

**DOI:** 10.1002/advs.202523928

**Published:** 2026-02-09

**Authors:** Luyi Wang, Jiasha Wu, Rui Ji, Sufeng Qiang, Yulin Shen, Yan Zuo, Shiqin Jian, Siyao Liu, Fusheng Xu, Honggang Hu, Xiaochun Hu

**Affiliations:** ^1^ School of Medicine Shanghai Integration and Innovation Center of Marine Medical Engineering Shanghai University Shanghai China; ^2^ Department of Chemistry College of Sciences Shanghai University Shanghai China; ^3^ Department of Gynaecology and Obstetrics School of Medicine Shanghai East Hospital Tongji University Shanghai China; ^4^ School of Pharmacy Chengdu Medical College Chengdu China

**Keywords:** BRD4, cancer immunotherapy, metal‐organic frameworks, photothermal degradation, theranostics

## Abstract

Conventional targeted protein degradation (TPD) via PROTACs is limited by E3 ligase reliance and a lack of spatiotemporal control, often causing systemic toxicity. Here, we report a photothermolysis‐targeting conjugate (PTTAC) strategy for precise, light‐activated degradation. We constructed a multifunctional theranostic nanoplatform (PCN‐CuS‐JQ/RGD) based on an MRI‐visible, Fe‐porphyrin MOF (PCN(Fe)) carrier, decorated with CuS photothermal agents, a BRD4 inhibitor (JQ1), and a tumor‐targeting peptide (RGD). It executes a sophisticated step‐wise delivery: 1) The RGD peptide facilitates active targeting to integrin‐overexpressing tumor cells. 2) Upon internalization, the PCN(Fe) carrier decomposes within the cell, releasing smaller CuS‐JQ/RGD units. 3) These units enter the nucleus and bind to the BRD4 protein. Under 1064 nm (NIR‐II) laser irradiation, the localized photothermal effect generated by CuS induces the efficient degradation of BRD4. This PTTAC strategy not only inhibits tumor growth but also initiates a robust anti‐tumor immune response by inducing immunogenic cell death (ICD) and downregulating PD‐L1 expression. Crucially, in a bilateral tumor‐bearing mouse model, our strategy demonstrated a powerful synergistic effect, significantly enhancing the efficacy of anti‐PD‐L1 immune checkpoint blockade therapy. This work presents a light‐activatable degradation platform that achieves high tumor selectivity and potent immunotherapy, offering a promising new avenue for cancer treatment.

## Graphical abstract

A GSH‐responsive MOF nanoplatform acts as a Photothermolysis‐Targeting Conjugate (PTTAC). Through sophisticated step‐wise nuclear delivery, it enables NIR‐II light‐triggered photothermal degradation of BRD4. This precise strategy induces robust immunogenic cell death (ICD) and powerfully synergizes with anti‐PD‐L1 blockade to elicit a potent systemic anti‐tumor immune response.

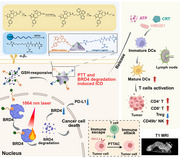

## 1 Introduction

Targeted protein degradation (TPD), particularly via Proteolysis‐Targeting Chimeras (PROTACs), has emerged as a revolutionary therapeutic modality. This strategy co‐opts the ubiquitin‐proteasome system (UPS) by utilizing heterobifunctional molecules to recruit a specific E3 ligase, thereby ubiquitinating and degrading a protein of interest (POI) [1]. Despite its transformative potential, the clinical translation of PROTACs faces significant hurdles. Firstly, the development of PROTACs is heavily dependent on a very small subset of the > 600 known human E3 ligases, most notably CRBN and VHL [2]. This limited “toolbox” not only restricts the scope of degradable proteins but also fosters acquired resistance through mechanisms like E3 ligase mutation or downregulation [3]. Secondly, as systemically administered small molecules, conventional PROTACs lack spatiotemporal control. This lack of tissue selectivity can lead to significant systemic toxicity, as the target protein is degraded in healthy tissues as well as in the tumor [4]. Therefore, there is an urgent need for novel TPD strategies that can bypass the reliance on specific E3 ligases and offer precise, controllable degradation, especially within the tumor microenvironment.

Light‐based therapies, including photodynamic therapy (PDT) [5] and photothermal therapy (PTT), are clinically established modalities renowned for their exceptional spatiotemporal precision. The therapeutic effect is confined only to the area and time of laser irradiation, offering minimal systemic toxicity [6, 7]. Leveraging this high‐precision control for protein degradation emerges as a highly attractive strategy. To date, the majority of research in this area has focused on PDT‐mediated protein degradation, where light‐induced reactive oxygen species (ROS) can induce protein oxidation, unfolding, and subsequent breakdown [8, 9]. In contrast, the exploration of photothermal‐mediated protein degradation, which employs localized hyperthermia to denature and degrade proteins, remains in its infancy. Pioneering studies by Tang's group [10] and others [11, 12] have recently validated the theoretical feasibility of photothermal‐mediated degradation. The development of such PTT systems hinges on the selection of an efficient photothermal agent (PTA). While various PTAs, including noble metal nanomaterials, carbon‐based materials, and semiconductor nanocrystals, have been investigated, Copper Sulfide (CuS) nanoparticles have attracted considerable attention. CuS is widely acknowledged for its high photothermal conversion efficiency (PCE), excellent biocompatibility, and, crucially, strong absorption and stability within the second near‐infrared (NIR‐II) window (1000‐1700 nm) [13, 14]. The utilization of NIR‐II light is of paramount importance for in vivo applications, as it offers superior tissue penetration depth and a higher maximum permissible exposure in comparison to conventional NIR‐I light [15]. Consequently, the development of a CuS‐based, NIR‐II‐activated PTT degradation system that can be precisely targeted to tumors holds profound therapeutic potential.

**SCHEME 1:**
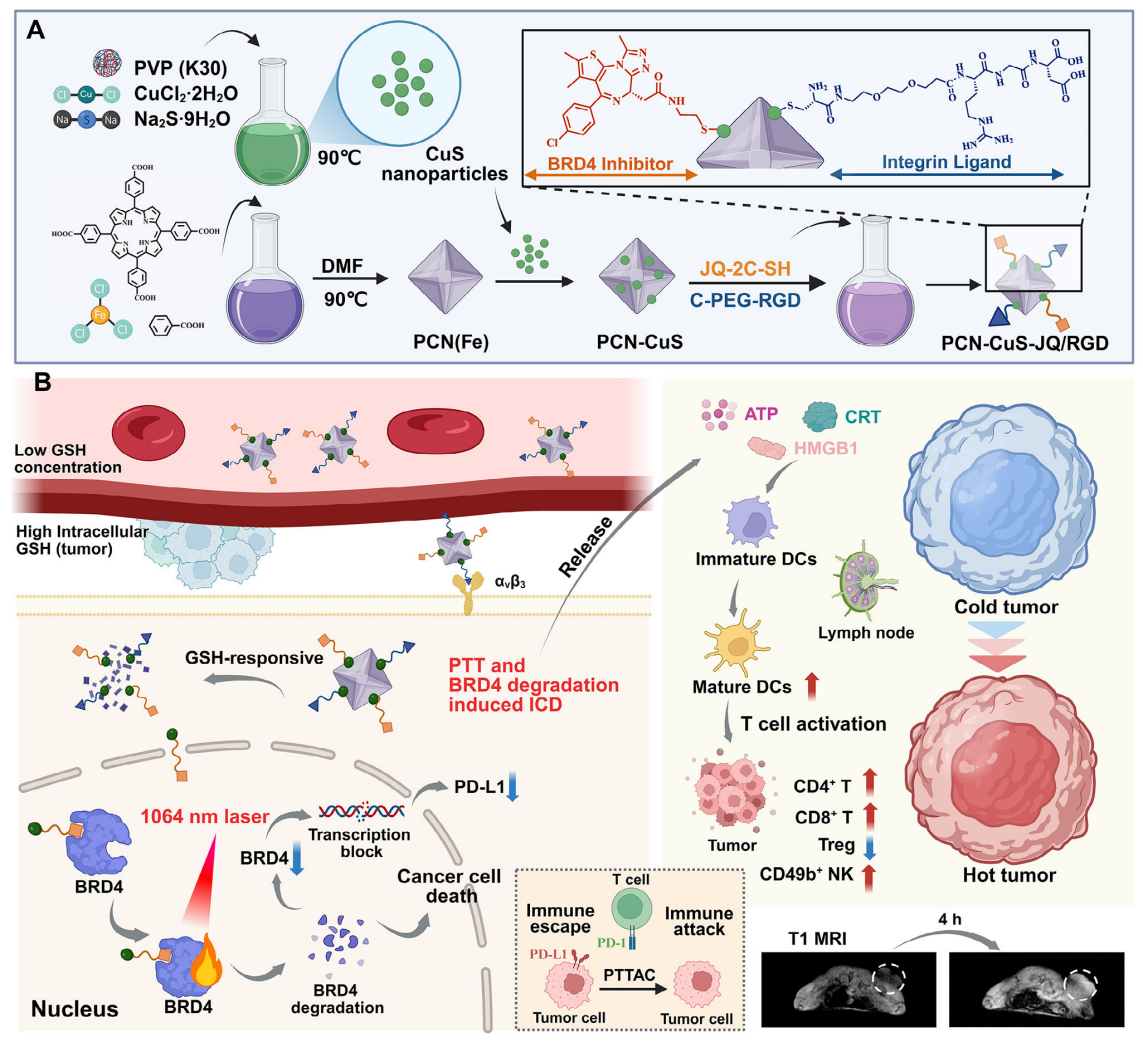
Fabrication procedures and antitumor mechanisms of PCN‐CuS‐JQ/RGD. (A) Schematic representation of the synthesis of PCN‐CuS‐JQ/RGD. (B) Anti‐tumor schematic diagram of the PCN‐CuS‐JQ/RGD.

Herein, we selected Bromodomain‐containing protein 4 (BRD4) as the therapeutic target. As a critical epigenetic reader, BRD4 plays a central role in tumorigenesis by driving the transcription of key oncogenes, such as MYC [16]. Moreover, emerging evidence implicates BRD4 in immune evasion through its regulation of the immune checkpoint ligand PD‐L1 [17]. This dual role makes BRD4 an exceptionally valuable target. To achieve a highly controlled, tumor‐specific degradation of BRD4, a multifunctional photothermolysis‐targeting conjugate (PTTAC) nanoplatform, designated PCN‐CuS‐JQ/RGD, was designed and constructed (Scheme 1A). The system utilizes a Fe‐porphyrin metal‐organic framework (PCN(Fe)) as a responsive carrier, which also enables magnetic resonance imaging (MRI) for in vivo tracking. The platform is functionalized with CuS (a NIR‐II PTA), JQ1 (a BRD4 ligand) [18], and RGD (a tumor‐targeting peptide). This design facilitates a step‐wise delivery sequence: the RGD peptide first guides the nanoplatform to tumor cells, and upon internalization, the PCN(Fe) carrier responsively decomposes. This decomposition subsequently releases the smaller CuS‐JQ/RGD units, which are then able to enter the nucleus and bind to BRD4. This cascade achieves a highly localized accumulation at the target site, setting the stage for NIR‐II light‐triggered photothermal degradation. Beyond direct tumor inhibition, this work reveals that PTTAC‐mediated BRD4 degradation effectively reshapes the tumor immune microenvironment, notably by triggering immunogenic cell death (ICD) and suppressing PD‐L1 expression. The potent immunomodulatory effect culminates in a powerful synergy with anti‐PD‐L1 (aPD‐L1) checkpoint blockade, significantly amplifying the therapeutic response in a bilateral tumor model and offering a robust strategy for combined cancer immunotherapy (Scheme 1B).

## 2 Results and Discussion

### 2.1 Preparation and Characterization of PCN‐CuS‐JQ/RGD

**FIGURE 1:**
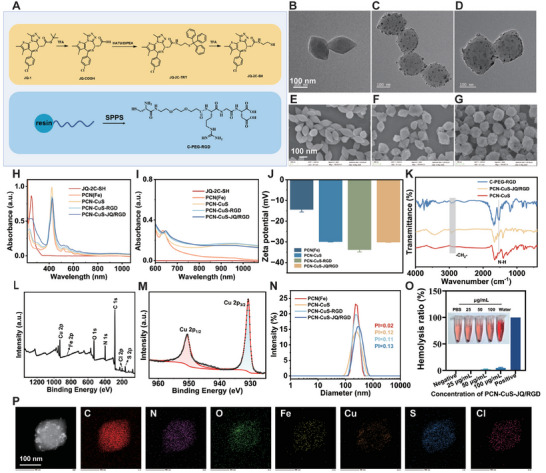
Synthesis and characterization of the nanoparticles. (A) The synthesis procedure of the BRD4 inhibitor (JQ‐2C‐SH) and the tumor‐targeted peptide (C‐PEG‐RGD). (B–D) TEM and (E–G) SEM images of PCN(Fe), PCN‐CuS, and PCN‐CuS‐JQ/RGD, respectively. (H) Full‐range (220–1070 nm) and (I) enlarged NIR‐region (600–1070 nm) UV–vis absorption spectra of JQ‐2C‐SH, PCN(Fe), PCN‐CuS, PCN‐CuS‐RGD, and PCN‐CuS‐JQ/RGD. (J) Zeta potentials and (N) hydrodynamic size distributions of the nanoparticles at different modification stages. (K) FTIR spectra of C‐PEG‐RGD, PCN‐CuS, and PCN‐CuS‐JQ/RGD. (L) XPS survey spectrum and (M) high‐resolution Cu 2p spectrum of PCN‐CuS‐JQ/RGD. (O) Hemolysis assay of PCN‐CuS‐JQ/RGD at various concentrations (using water as a positive control). (P) Elemental mapping images of PCN‐CuS‐JQ/RGD. Data are presented as mean ± s.e.m. (*n* = 3).

The synthesis pathway for the multifunctional nanoplatform, PCN‐CuS‐JQ/RGD, is depicted in Scheme 1A. Initially, the functional ligands, the BRD4 inhibitor (JQ‐2C‐SH) and the tumor‐targeting peptide (C‐PEG‐RGD), both of which possess terminal thiol groups, were prepared (Figure 1A). The JQ‐2C‐SH derivative was synthesized via a three‐step chemical reaction (de‐Boc, condensation, and de‐Trt), with each step's product structurally verified by ^1^H‐NMR, ^13^C‐NMR, and High Resolution Mass Spectrometry (HRMS) (Figures S1–S9). The C‐PEG‐RGD was synthesized by standard solid‐phase peptide synthesis (SPPS) and confirmed by High Performance Liquid Chromatography (HPLC) and HRMS (Figures S10, S11 and Table S1). Subsequently, the synthesis of PCN(Fe) and CuS nanoparticles was conducted separately under elevated temperature conditions. The CuS nanoparticles were then integrated onto the PCN(Fe) carrier via electrostatic adsorption. Finally, in a mixed solvent of water and methanol, the BRD4 inhibitor and the RGD peptide were coordinated and loaded onto the CuS nanoparticles through their terminal thiol groups [19].

Transmission electron microscopy (TEM) (Figure 1B–D) and scanning electron microscopy (SEM) (Figure 1E–G) images revealed that the PCN(Fe) carrier possessed a distinct granular surface after CuS adsorption. The UV–vis spectrum (Figure 1I) of PCN‐CuS‐JQ/RGD showed an absorption at 1064 nm, indicating the successful adsorption of CuS nanoparticles. Since the CuS nanoparticles carry a negative surface charge [20], their adsorption resulted in a more negative Zeta potential for the entire nanosystem (Figure 1J). Compared to PCN‐CuS, the FTIR spectrum (Figure 1K) of PCN‐CuS‐JQ/RGD displayed a large increase in the absorption bands corresponding to the asymmetric and symmetric stretching vibrations of the methylene groups (2932, 2878 cm^−1^)^[^ ^21^ ^]^ and the amide N‐H bending vibration (amide II) (1540 cm^−1^) [22]. These peaks matched the characteristic signatures of C‐PEG‐RGD, confirming the successful loading of the PEG‐modified tumor‐targeting peptide. The UV–vis spectrum (Figure 1H) showed the characteristic absorption peak of the JQ‐2C‐SH within PCN‐CuS‐JQ/RGD. Additionally, X‐ray photoelectron spectroscopy (XPS) analysis (Figure 1L and Figure S12) and elemental mapping (Figure 1P) detected the presence of chlorine (Cl), a characteristic element found only in the JQ‐2C‐SH. These data collectively confirm the successful loading of the JQ‐2C‐SH. In the XRD pattern (Figure S13), a peak observed around 48° corresponded to the diffraction peak of CuS [20], further verifying the successful adsorption of the CuS nanoparticles with XPS analysis (Figure 1M). Dynamic Light Scattering (DLS) measurements (Figure 1N) showed that the PCN‐CuS‐JQ/RGD system has a hydrodynamic size of approximately 280 nm, and its polydispersity index (PI) is 0.13, confirming its good uniform dispersion. Collectively, the above data confirm the successful synthesis of the PCN‐CuS‐JQ/RGD nanoplatform. As hemocompatibility is a prerequisite for in vivo application, a hemolysis assay was conducted. Even at a high concentration of 100 µg/mL, the hemolysis rate of PCN‐CuS‐JQ/RGD remained below 5%. This result confirms the high hemocompatibility of the nanosystem, strongly supporting its potential for safe in vivo administration (Figure 1O).

### 2.2 Photothermal Performance and Stimuli‐Responsive Behavior

**FIGURE 2:**
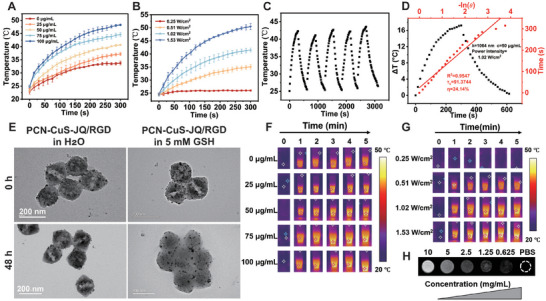
The properties of PCN‐CuS‐JQ/RGD. (A) Temperature elevation curves and (F) infrared thermal images of PCN‐CuS‐JQ/RGD aqueous suspensions at varying concentrations (0, 25, 50, 75, and 100 µg/mL) under the 1064 nm laser irradiation (1.02 W/cm^2^) for 5 min. (B) Temperature elevation curves and (G) thermal images of PCN‐CuS‐JQ/RGD (50 µg/mL) at different laser power densities (0.25, 0.51, 1.02, and 1.53 W/cm^2^). (C) Photothermal stability evaluation of PCN‐CuS‐JQ/RGD over five laser on/off cycles (50 µg/mL, 1.02 W/cm^2^). (D) Linear fitting of the cooling time versus *‐ln(θ)* for calculating the PCE. (E) TEM images showing the structural integrity of PCN‐CuS‐JQ/RGD (2 mg/mL) after incubation in water (left) versus its decomposition in 5 mm GSH (right) for 48 h. (H) *T*_1_‐weighted MRI phantom images of PCN‐CuS‐JQ/RGD at different concentrations (0–10 mg/mL). Data are presented as mean ±  s.e.m. (*n* = 3).

The photothermal properties of PCN‐CuS‐JQ/RGD were then evaluated. The temperature change of the nanosystem over time was investigated under 1064 nm laser irradiation at varying concentrations and power densities (Figure 2A, B). Notably, when the PCN‐CuS‐JQ/RGD solution (75 µg/mL) was irradiated at a power density of 1.02 W/cm^2^, the temperature reached approximately 45°C after 5 min, which was also visualized by thermal imaging (Figure 2F, G). This is particularly significant, as the 42°C‐45°C range is widely considered the critical “hyperthermia window” for therapy. Achieving 45°C confirms the nanosystem can reach this therapeutic threshold. This localized hyperthermia is not only sufficient to induce tumor cell death but is also known to cause protein unfolding, denaturation, and aggregation [12, 23], which provides the precise mechanism for the photothermal‐mediated protein degradation investigated in this study. To verify its photothermal stability, the PCN‐CuS‐JQ/RGD solution was subjected to five repeated heating‐cooling cycles. The nanoplatform maintained a stable photothermal conversion capability without significant decay (Figure 2C), indicating its excellent thermal stability for PTT applications. The photothermal conversion efficiency (PCE) of PCN‐CuS‐JQ/RGD was calculated to be 24.14% (Figure 2D and Figure S14), confirming its high efficiency as a NIR‐II PTA.

A key feature of our design is the “step‐wise” delivery, which requires the PCN(Fe) carrier to decompose upon entering the tumor cell, releasing the smaller, functional CuS‐JQ/RGD units. We hypothesized this decomposition would be triggered by the high intracellular glutathione (GSH) concentration (up to 10 mm) in the tumor microenvironment (TME) [24]. To validate this, PCN‐CuS‐JQ/RGD was incubated in either pure water or a 5 mm GSH solution (simulating the TME) for 48 h. The TEM images (Figure 2E) indicated that in pure water, the nanoplatform remained intact, retaining its large PCN(Fe) framework structure. In contrast, after incubation in 5 mm GSH, the PCN(Fe) framework showed clear evidence of structural collapse and decomposition. This GSH‐triggered transformation of the nanosystem from a large nanocarrier into its smaller constituent nanoparticles is a critical design feature. This size reduction is essential for the components to translocate through the nuclear pore, target the nucleus‐localized BRD4 protein, and enable the subsequent photothermal degradation. Furthermore, the constituent paramagnetic ferric ions (Fe^3^^+^) within the PCN(Fe) framework endow the PCN‐CuS‐JQ/RGD nanoplatform with *T*_1_‐weighted magnetic resonance imaging (MRI) capability [25]. In vitro experiments (Figure 2H) demonstrated a concentration‐dependent increase in signal intensity on *T*_1_‐weighted MRI scans, confirming its potential for MRI‐guided antitumor therapy.

### 2.3 In Vitro Antitumor Effect of PTTAC Strategy

**FIGURE 3:**
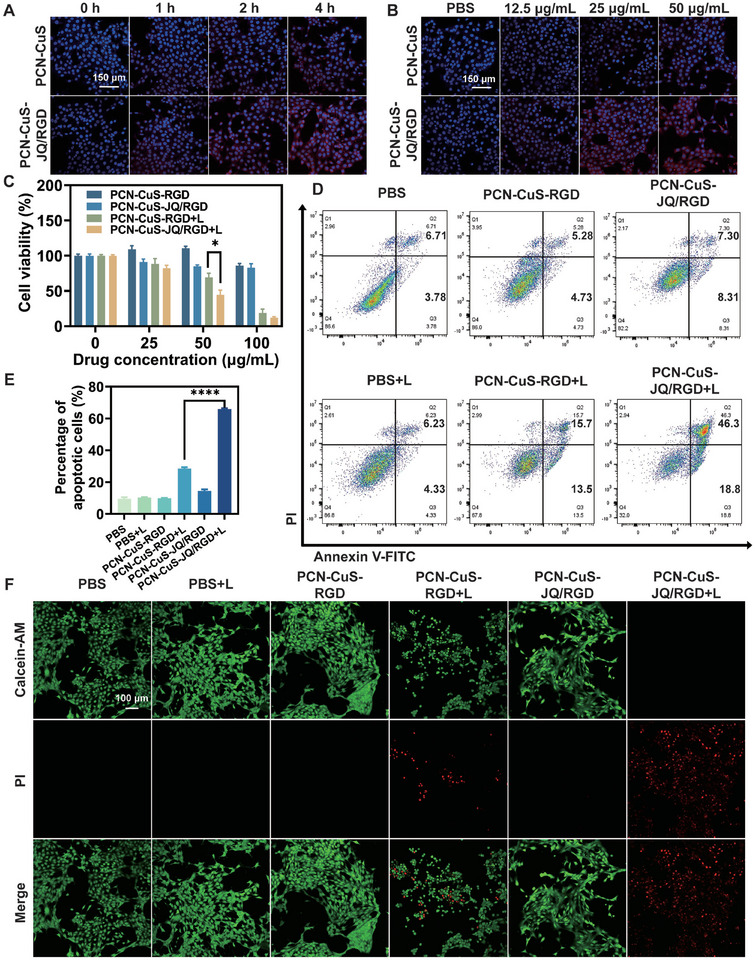
In vitro cellular uptake and antitumor efficacy of PCN‐CuS‐JQ/RGD. CLSM images showing the cellular uptake and intracellular distribution of Rhodamine B‐labeled PCN‐CuS and PCN‐CuS‐JQ/RGD in 4T1 cells at different incubation times (A) and concentrations (B). (C) Cytotoxicity of PCN‐CuS‐RGD and PCN‐CuS‐JQ/RGD (with or without laser irradiation) against 4T1 cells assessed by CCK8 assay. Flow cytometric analysis (D) and corresponding quantitative analysis (E) of apoptosis in 4T1 cells following different treatments. (F) Live/dead staining (Calcein‐AM/PI) of 4T1 cells after indicated treatments. Data are presented as mean ±  s.e.m. (*n* = 3). ^*^ *p* < 0.05, ^****^ *p* < 0.0001.

Prior to evaluating the therapeutic efficacy, the cellular uptake of the nanoplatform was verified. Confocal laser scanning microscopy (CLSM) images of murine breast cancer cells (4T1) incubated with Rhodamine B‐labeled PCN‐CuS or PCN‐CuS‐JQ/RGD revealed a distinct time‐ and concentration‐dependent uptake pattern (Figure 3A, B). Notably, the PCN‐CuS‐JQ/RGD group exhibited a significantly enhanced cellular internalization compared to the unmodified PCN‐CuS, likely attributed to RGD‐mediated targeting. Subsequently, the in vitro cytotoxicity of PCN‐CuS‐JQ/RGD and PCN‐CuS‐RGD against 4T1 cells was assessed using the Cell Counting Kit‐8 (CCK‐8) assay. After incubating 4T1 cells with varying concentrations of PCN‐CuS‐JQ/RGD or PCN‐CuS‐RGD for 24 h, the cells were irradiated with a 1064 nm laser for 5 min. Cell viability was measured 24 h post‐irradiation. As shown in Figure 3C, neither PCN‐CuS‐JQ/RGD nor PCN‐CuS‐RGD exhibited significant toxicity toward 4T1 cells in the absence of laser irradiation. However, upon laser exposure, cell viability in the group treated with PCN‐CuS‐JQ/RGD at 50 µg/mL decreased significantly to below 50%. To assess potential side effects on normal cells, CCK‐8 assays were performed on 293T and 3T3 cell lines (Figure S15). The results showed no significant cytotoxicity, indicating good biocompatibility and supporting its safety profile in vitro.

To further investigate the anti‐tumor mechanism, the apoptosis levels induced by PCN‐CuS‐JQ/RGD (50 µg/mL) under 1064 nm laser irradiation were examined. Live/dead cell staining was performed using Calcein‐AM/PI. As expected, the combination of PCN‐CuS‐JQ/RGD and laser irradiation caused extensive cell death in 4T1 cells, whereas only limited cell death was observed in the PCN‐CuS‐RGD+L group (Figure 3F). This indicates that PCN‐CuS‐JQ/RGD elicits severe cytotoxicity specifically in the presence of laser irradiation. Furthermore, flow cytometry analysis confirmed a statistically significant increase in apoptosis (Figure 3D, E). Notably, following irradiation with a 1064 nm laser at 2.04 W/cm^2^ for 5 min, the apoptotic cell rate in the PCN‐CuS‐JQ/RGD group increased from 10.5% to 65.1%. Collectively, these results demonstrate that PCN‐CuS‐JQ/RGD possesses a precisely light‐controllable and pronounced synergistic anti‐tumor efficacy in vitro.

### 2.4 Light‐Triggered BRD4 Degradation and ICD Induction in Living Cells

**FIGURE 4:**
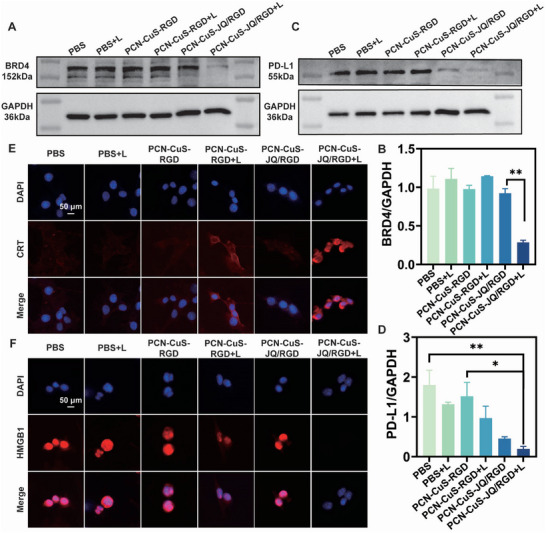
In vitro validation of PTTAC‐mediated BRD4 degradation and ICD induction in 4T1 cells. Western blot images (A) and quantitative analysis (B) of BRD4 expression in 4T1 cells after indicated treatments. Western blot images (C) and quantitative analysis (D) of PD‐L1 expression under various treatment conditions. Immunofluorescence staining images of CRT (E) and HMGB1 (F) levels in 4T1 cells after diverse treatments. Data are presented as mean ±  s.e.m. (*n* = 3). ^*^ *p* < 0.05, ^**^ *p* < 0.01.

To investigate the light‐triggered protein degradation capability, the expression of BRD4 in 4T1 cells was assessed following treatment with PCN‐CuS‐JQ/RGD and laser irradiation. Western blot analysis (Figure 4A, B) demonstrated that BRD4 protein levels were significantly downregulated only upon laser irradiation in the presence of PCN‐CuS‐JQ/RGD. This observation confirms that the PCN‐CuS‐JQ/RGD nanoplatform specifically binds to the BRD4 protein without inducing degradation in the absence of light; instead, effective degradation is achieved predominantly via the photothermal effect induced by 1064 nm laser irradiation. Furthermore, given that specifically degrading BRD4 in tumor cells can significantly reduce PD‐L1 expression, thereby enhancing the anti‐tumor immune response [26], the expression of PD‐L1 was also evaluated (Figure 4C, D). A significant downregulation of PD‐L1 was observed after treatment with PCN‐CuS‐JQ/RGD combined with laser irradiation. Of note, a substantial decrease in PD‐L1 was also evident in the non‐irradiated PCN‐CuS‐JQ/RGD group. Although the JQ1 moiety within the nanoplatform does not degrade BRD4 directly, it is known to inhibit BRD4 recruitment at the *CD274* promoter region, since *CD274* is a direct target gene of BRD4 [17]. Therefore, the observed PD‐L1 reduction in the non‐irradiated group is likely attributed to JQ1‐mediated transcriptional inhibition.

Additionally, both the downregulation of BRD4 and the application of PTT can activate ICD [27, 28]. Unlike immunologically silent apoptosis, ICD is characterized by the specific release of damage‐associated molecular patterns (DAMPs) during cell death [29]. Here, the cell surface exposure of calreticulin (CRT) and the nuclear release of high mobility group box 1 (HMGB1) were examined. Immunofluorescence staining revealed a significant increase in CRT exposure (Figure 4E) and a marked reduction in nuclear HMGB1 levels (Figure 4F) in the PCN‐CuS‐JQ/RGD+L group compared to the control. These results confirm that PCN‐CuS‐JQ/RGD, when activated by laser irradiation, can effectively induce ICD.

### 2.5 In Vivo Biodistribution of PCN‐CuS‐JQ/RGD

**FIGURE 5:**
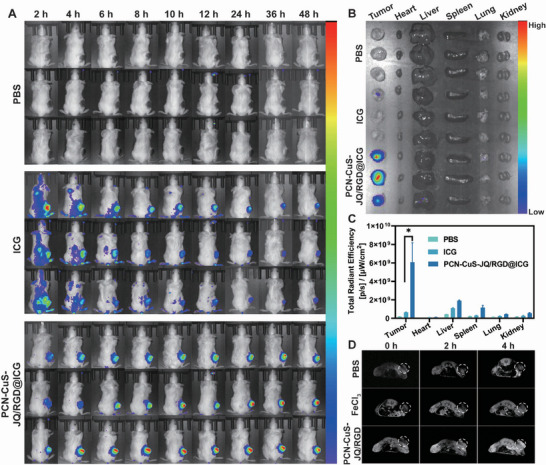
In vivo tumor targeting efficiency and MRI capability. (A) In vivo fluorescence imaging of 4T1‐tumor‐bearing mice at different time points after intravenous injection of PBS, free ICG, or PCN‐CuS‐JQ/RGD@ICG. *Ex vivo* fluorescence imaging (B) and semiquantitative analysis (C) of tumors and major organs harvested at 48 h post‐injection. (D) In vivo time‐dependent *T*_1_‐weighted MRI after intravenous injection of PBS, FeCl_3_, or PCN‐CuS‐JQ/RGD. Data are presented as mean ±  s.e.m. (*n* = 3). ^*^ *p* < 0.05.

To investigate the in vivo tumor‐targeting efficiency and biodistribution, near‐infrared (NIR) fluorescence imaging was utilized. The fluorescent dye indocyanine green (ICG) was encapsulated within the nanoplatform to create PCN‐CuS‐JQ/RGD@ICG. 4T1 tumor‐bearing mice were intravenously injected with PBS, free ICG, or PCN‐CuS‐JQ/RGD@ICG. Compared to mice injected with free ICG, those receiving PCN‐CuS‐JQ/RGD@ICG exhibited a significantly stronger fluorescence signal at the tumor site (Figure 5A), indicating superior in vivo tumor‐targeting and retention capabilities. The mice were sacrificed 48 h post‐injection, *ex vivo* fluorescence images (Figure 5B) and statistical analysis (Figure 5C) of the tumors and major organs were acquired. The results were consistent with the in vivo imaging data, corroborating the excellent tumor‐targeting and retention capabilities of the nanosystem.

Building on this validated tumor accumulation, the in vivo *T*_1_‐weighted MRI capability of PCN‐CuS‐JQ/RGD, attributed to the inherent paramagnetic Fe^3^^+^ within the PCN(Fe) framework, was assessed. Tumor‐bearing mice were intravenously injected with PBS, ferric chloride (FeCl_3_), or PCN‐CuS‐JQ/RGD. A significant, time‐dependent enhancement of the *T*_1_ signal intensity was observed in the tumor region of the PCN‐CuS‐JQ/RGD group, while no significant contrast change was seen in the control groups (Figure 5D). Taken together, these results demonstrate that PCN‐CuS‐JQ/RGD functions as an effective *T*_1_‐weighted MRI contrast agent, capable of visualizing tumor accumulation in vivo.

### 2.6 In Vivo Antitumor Efficacy of PTTAC Strategy

**FIGURE 6:**
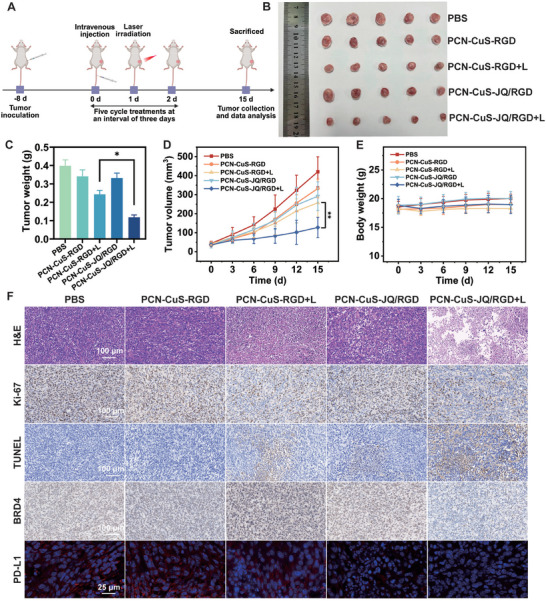
In vivo therapeutic efficacy. (A) Schematic illustration of the therapeutic regimen. (B) Photographs of tumors excised from mice at the end of the treatment. (C) Tumor weights of mice after indicated treatments. (D) Tumor growth curves of mice during the treatment period. (E) Body weight changes of mice were monitored over 15 days. (F) H&E staining, Ki‐67 staining, TUNEL staining, BRD4 immunohistochemical staining, and PD‐L1 immunofluorescence staining of tumor tissues after different treatments. Data are presented as mean ±  s.e.m. (*n* = 5). ^*^ *p* < 0.05, ^**^ *p* < 0.01.

Encouraged by the promising in vitro cytotoxicity and proven tumor accumulation, the in vivo anti‐tumor efficacy of the PTTAC strategy was evaluated. The photothermal performance of PCN‐CuS‐JQ/RGD in vivo was confirmed by thermal imaging (Figure S16), showing a rapid temperature increase from approximately 36°C to 45°C within 5 min, which is sufficient for inducing both hyperthermic damage and widespread protein denaturation. A 4T1 tumor‐bearing mouse model was established and randomly divided into five groups: (1) PBS control, (2) PCN‐CuS‐RGD, (3) PCN‐CuS‐RGD+L, (4) PCN‐CuS‐JQ/RGD, and (5) PCN‐CuS‐JQ/RGD+L. Nanomaterials were administered via tail vein injection, and laser irradiation (1064 nm, 5 min) was applied at 24 h post‐injection. This treatment cycle was repeated every three days for five cycles (Figure 6A). Throughout the treatment period, tumor volume and mouse body weight were recorded every three days. The groups without laser irradiation (PBS, PCN‐CuS‐RGD, and PCN‐CuS‐JQ/RGD) exhibited rapid and uncontrolled tumor growth, confirming that the nanoplatforms possess negligible dark cytotoxicity and require laser activation for therapy. The PTT‐only group (PCN‐CuS‐RGD+L) showed moderate tumor suppression. Remarkably, the PTTAC group (PCN‐CuS‐JQ/RGD+L) achieved potent tumor inhibition and was significantly more effective than the PTT‐only group (Figure 6B–D). This result provides powerful evidence for the in vivo synergistic effect of combining PTT with targeted BRD4 degradation. Furthermore, no significant fluctuations in body weight were observed in the treatment groups compared to the PBS control (Figure 6E), indicating high systemic tolerability. Blood biochemistry and histological evaluations of major organs (Figures S17–S19) revealed no obvious abnormalities, further confirming the biosafety of the combined application of PCN‐CuS‐JQ/RGD and 1064 nm laser irradiation in vivo.

To elucidate the therapeutic mechanism at the tissue level, tumors were collected after treatment for histological analysis (Figure 6F). H&E staining images revealed that the PTTAC group exhibited extensive areas of necrosis compared to other treatment groups. Consistent with this, Ki‐67 staining showed the lowest proliferation activity, while TUNEL staining indicated the highest percentage of apoptotic cells in this group. Crucially, immunohistochemical analysis was performed to assess BRD4 protein expression. As anticipated, the PTTAC group demonstrated a marked downregulation of BRD4 protein levels, whereas no significant variations were observed in the other groups, confirming the successful photothermal‐mediated degradation in vivo. In parallel, immunofluorescence analysis of PD‐L1 yielded consistent results: while the non‐irradiated PCN‐CuS‐JQ/RGD group showed a moderate decrease in PD‐L1 levels (due to transcriptional inhibition), the PTTAC group exhibited the most pronounced suppression. These results collectively demonstrate that the PTTAC strategy effectively inhibits tumor growth in vivo through a light‐controlled, synergistic mechanism involving BRD4 degradation and PD‐L1 suppression.

### 2.7 In Vivo Antitumor Immune Response

**FIGURE 7:**
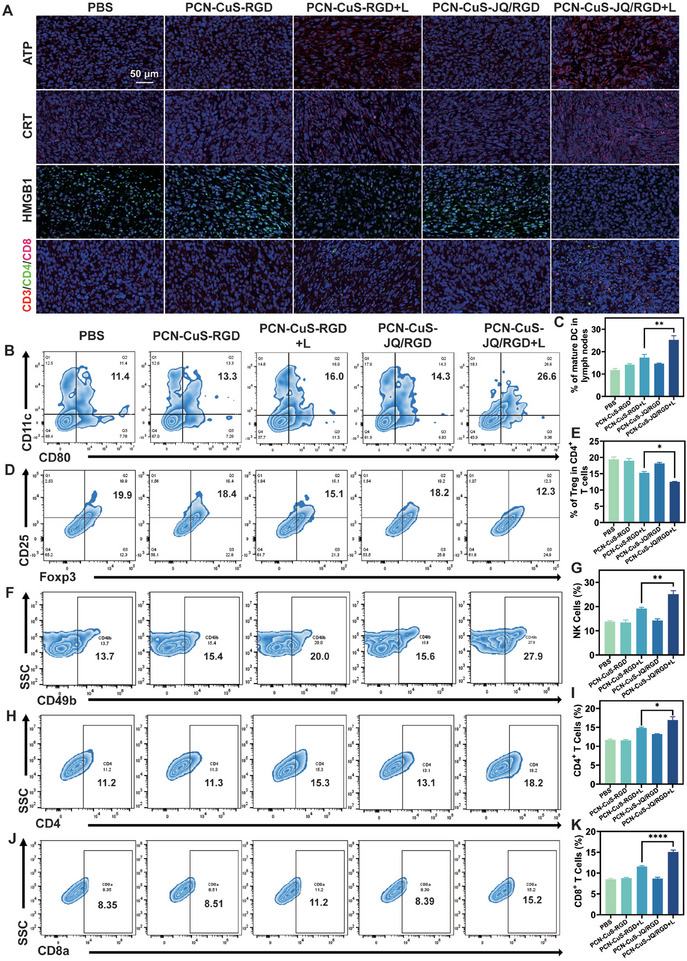
Activation of systemic antitumor immunity and remodeling of the tumor microenvironment. (A) Immunofluorescence staining of ICD markers (ATP, CRT, HMGB1) and T lymphocytes (CD3, CD4, CD8) in tumor tissues after different treatments. Flow cytometry plots (B) and quantification (C) of mature dendritic cells in lymph nodes. Flow cytometric assay (D) and percentages (E) of Tregs in tumors. Flow cytometry plots (F) and quantification (G) of NK cells in tumors. Flow cytometric assay (H) and percentages (I) of CD4^+^ T cells in tumors. Flow cytometry plots (J) and quantification (K) of CD8a^+^ T cells in tumors after different treatments. Data are presented as mean ± s.e.m. (*n* = 3). ^*^ *p* < 0.05, ^**^ *p* < 0.01, ^****^ *p* < 0.0001.

As mentioned above, PCN‐CuS‐JQ/RGD selectively degrades BRD4 under laser irradiation, and the application of PTT synergistically generates an anti‐tumor immune response. To elucidate the immune mechanisms underlying the potent in vivo efficacy of the PTTAC strategy, immunofluorescence analysis was performed on tumors from mice in each group on day 15 post‐treatment (Figure 7A). The results showed that the PTTAC group exhibited a significant increase in ATP secretion and CRT exposure, accompanied by a notable loss of nuclear HMGB1, compared to all the other groups. While the PTT‐only group also demonstrated a response, it was substantially weaker than that of the PTTAC group, corroborating the synergistic role of BRD4 degradation in promoting ICD. Furthermore, tumor immunofluorescence staining revealed a substantial increase in CD3^+^, CD4^+^, and CD8^+^ T cells within the tumor sites of the PTTAC group. This indicates that the combination of PCN‐CuS‐JQ/RGD and laser irradiation facilitates the recruitment and function of cytotoxic T cells and effector T cells in the tumor, thereby enhancing the anti‐tumor immune response. These findings were further quantitatively validated by flow cytometric analysis (Figure 7H–K).

To further delineate the systemic immune activation, the maturation of dendritic cells (DCs) and the status of immunosuppressive cells were analyzed. Compared to other groups, the PTTAC group showed a significant increase in the population of mature DCs (CD11c^+^/CD80^+^) in the lymph nodes (Figure 7B, C). This suggests enhanced antigen presentation and T‐cell priming, a critical step for initiating the systemic immune response [30]. Furthermore, the frequency of regulatory T cells (Tregs, CD4^+^/CD25^+^/Foxp3^+^), which act to suppress anti‐tumor immunity, was assessed [31]. The results demonstrated that the PTTAC group had a significantly reduced frequency of Tregs compared to the other groups, confirming a significant reduction in tumor immune suppression in this group (Figure 7D, E). Additionally, the contribution of the innate immune system was evaluated by quantifying Natural Killer (NK) cells, which serve as crucial innate effectors [32]. In mice, CD49b (α2 integrin chain) widely serves as a pan‐NK cell marker [33]. Flow cytometry analysis (Figure 7F, G) revealed that the PTTAC group had the highest number of NK cells (CD3^−^/CD49b^+^). To further characterize the activation status of these infiltrating NK cells, immunofluorescence staining (Figure S20) for NKp46 and Granzyme B (GZMB) was performed [34]. NKp46 marks NK specificity [35], while GZMB signifies cytotoxic activity [36]. The imaging results indicated that NK cells in the PTTAC group not only exhibited enhanced infiltration into the tumor tissue but also achieved a higher level of functional activation. Taken together, these findings demonstrate that the PTTAC strategy not only directly kills tumor cells but also reshapes the tumor immune microenvironment, effectively enabling a potent and systemic anti‐tumor immunotherapy.

### 2.8 Synergistic Immunotherapy and Abscopal Effect in a Bilateral Tumor Model

**FIGURE 8:**
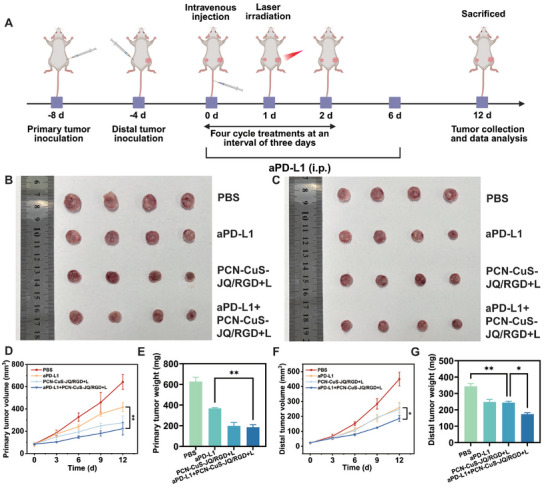
Abscopal effect and synergistic immunotherapy in a bilateral 4T1 tumor model. (A) Schematic illustration of the therapeutic regimen. Photographs of primary (B) and distal (distant) (C) tumors excised from mice after the indicated treatments. The growth curve of primary (D) and distal (F) tumors during the treatment period. Average weights of primary (E) and distal (G) tumors on day 12. Data are presented as mean ±  s.e.m. (*n* = 4). ^*^ *p* < 0.05, ^**^ *p* < 0.01.

Having established that our PTTAC strategy induces potent ICD and T‐cell infiltration (Section 2.7), we next sought to determine if this localized treatment could elicit a systemic anti‐tumor immune response and synergize with immune checkpoint blockade (ICB). Anti‐PD‐L1 antibody (aPD‐L1) was selected to alleviate immunosuppression and amplify the systemic response [37, 38]. A bilateral 4T1 tumor model was established, with a primary tumor on the right flank and a distant, non‐treated tumor on the left flank to simulate metastasis. These bilateral tumor‐bearing mice were randomly divided into four groups: (1) PBS, (2) aPD‐L1, (3) PCN‐CuS‐JQ/RGD+L (PTTAC), and (4) aPD‐L1+PCN‐CuS‐JQ/RGD+L (Combination). The mice received intravenous injections of PCN‐CuS‐JQ/RGD on days 0, 3, 6, and 9, with the laser groups receiving 1064 nm laser irradiation 24 h after each injection. Crucially, laser irradiation was applied only to the primary (right) tumor. Intraperitoneal injections of aPD‐L1 were administered on days 0 and 6 (Figure 8A). Tumor volumes on both sides were recorded every three days (Figure 8D, F). Mice were sacrificed on day 12, and tumors were collected, photographed, and weighed. In the primary (irradiated) tumors (Figure 8B, E), both the PTTAC group and the Combination group achieved substantial tumor suppression, with no significant difference between them. This indicates the local PTTAC treatment was already maximally effective. The most critical results were observed at the distant (non‐irradiated) tumor (Figure 8C, G). Compared to the PBS control, both the aPD‐L1 monotherapy and the PTTAC treatment (via the abscopal effect) achieved a moderate and statistically significant inhibition of tumor growth. Notably, there was no statistical difference between these two single‐treatment groups, suggesting they both provide a similar level of systemic immune response but reach a therapeutic plateau. In stark contrast, the Combination group demonstrated a markedly enhanced inhibition, which was statistically superior to both the aPD‐L1 alone and PTTAC‐only groups. These results provide compelling evidence for a powerful synergy. While either ICB or PTTAC‐mediated in situ immunization provides a significant benefit, they are not fully effective alone. Their combination, however, unlocks a statistically significant therapeutic advantage, allowing the checkpoint inhibitor to amplify the systemic anti‐tumor response and achieve enhanced control of distant, untreated metastases.

## 3 Conclusion

In summary, we have developed an innovative PTTAC strategy to overcome the intrinsic limitations of conventional PROTACs, specifically their reliance on a limited E3 ligase pool and lack of spatiotemporal control. We successfully constructed a theranostic nanoplatform, PCN‐CuS‐JQ/RGD, which integrates NIR‐II photothermal conversion, MRI guidance, and a sophisticated, GSH‐responsive step‐wise delivery mechanism. This system achieves highly precise, light‐activatable degradation of the nuclear protein BRD4 through localized hyperthermia. Crucially, this PTTAC strategy transcends mere tumor ablation. We demonstrated that the light‐triggered degradation of BRD4, in synergy with PTT, induces substantial ICD and effectively downregulates PD‐L1 expression. This process successfully reshapes the immunosuppressive tumor microenvironment, promoting the transition from cold to hot tumor phenotypes. Notably, this immunomodulation translates into a powerful, systemic anti‐tumor response, as evidenced by a significant abscopal effect. When combined with anti‐PD‐L1 checkpoint blockade, our PTTAC strategy exhibited a robust synergistic effect, leading to superior tumor suppression in a bilateral tumor model. Furthermore, the modular design of this platform is not limited to BRD4. By substituting the targeting ligand, this PTTAC concept offers a versatile platform with the potential to be adapted for a wide range of proteins, including membrane, nuclear, and cytoplasmic targets. Our work presents a novel, light‐mediated approach for precise protein degradation, offering a powerful and promising strategy for advancing cancer immunotherapy.

## Funding

This work was funded by the National Natural Science Foundation of China (22477075), the Fundamental Research Funds for the Central Universities (22120240343), and the General Project of Pudong New Area Health Commission Health Science and Technology Project (PW2024A‐14).

## Conflicts of Interest

The authors declare no conflicts of interest.

## Supporting information




**Supporting File**: advs74294‐sup‐0001‐SuppMat.docx

## Data Availability

The data that support the findings of this study are available from the corresponding author upon reasonable request.
