# Supplementary material for: A Spatiotemporally Controlled Nanoplatform for Photothermal BRD4 Degradation Enables Synergistic Cancer Immunotherapy
Source: Adv Sci (Weinh). 2026 Feb 9;13(22):e23928. doi: 10.1002/advs.202523928 (PMC13088268; doi:10.1002/advs.202523928)
Supplement: Supplementary file 1 — Supporting File: advs74294‐sup‐0001‐SuppMat.docx [file ADVS-13-e23928-s001.docx]

**Supporting Information**

**A Spatiotemporally Controlled Nanoplatform for Photothermal BRD4 Degradation Enables Synergistic Cancer Immunotherapy**

Luyi Wang, Jiasha Wu, Rui Ji, Sufeng, Qiang, Yulin Shen, Yan Zuo, Shiqin Jian, Siyao Liu, Fusheng Xu*, Honggang Hu*, Xiaochun Hu*

L. Wang, R. Ji, Y. Shen, Y. Zuo, S. Jian, S. Liu, F. Xu, H. Hu, X. Hu

School of Medicine, Shanghai Integration and Innovation Center of Marine Medical Engineering, Shanghai University, Shanghai 200444, China

E-mail: huxiaochun@shu.edu.cn; xufusheng@shu.edu.cn

J. Wu

Department of Chemistry, College of Sciences, Shanghai University, Shanghai 200444, China

S. Qiang

Department of Gynaecology and Obstetrics, Shanghai East Hospital, School of Medicine, Tongji University, Shanghai 200120, China

H. Hu

School of Pharmacy, Chengdu Medical College, Chengdu 610083, China

E-mail: hhu66@shu.edu.cn

**Experimental section**

**Chemicals and Materials.** (S)-Tert-Butyl 2-(4-(4-Chlorophenyl)-2,3,9-Trimethyl-6H-Thieno[3,2-f][1,2,4]Triazolo[4,3-a][1,4]Diazepin-6-yl)Acetate (JQ-1), Dichloromethane (DCM), 2-(7-Aza-1H-Benzotriazole-1-yl)-1,1,3,3-Tetramethyluronium Hexafluorophosphate (HATU), N,N-Diisopropylethylamine (DIPEA), N,N-Dimethylformamide (DMF), 2-(Tritylthio)Ethanamine, Triethylamine (TEA), Ethyl Acetate (EA), Methyl Alcohol (MeOH), Triisopropylsilane (TIPS), Fmoc-9-Amino-4,7-Dioxanonanoic Acid, 1-Hydroxybenzotriazole (HOBT), N,N'-Diisopropylcarbodiimide (DIC), Iron Chloride Hexahydrate (FeCl_3_·6H_2_O), Polyvinylpyrrolidone (PVP-K30), Copper(II) chloride dihydrate (CuCl_2_·2H_2_O) and Sodium sulfide nonahydrate (Na_2_S·9H_2_O) were purchased from Adamas Reagent, Co., Ltd. Trifluoroacetic Acid (TFA), Diisopropyl Ether (Ipe) and Piperidine were obtained from Sinopharm Chemical Reagent Co., Ltd (Shanghai, China). Benzoic acid was purchased from Bide Pharmatech Ltd (Shanghai, China). Tetrakis(4-Carboxyphenyl)Porphyrin (TCPP) was purchased from Shanghai Macklin Biochemical Co.,Ltd (Shanghai, China). Dulbecco’s Modified Eagle Medium (DMEM) and 4T1 cells (mouse breast cancer cells) were purchased from Wuhan Pricella Biotechnology Co., Ltd (Wuhan, China). Fetal bovine serum (FBS) and penicillin-streptomycin (PS) were obtained from Gibco. Cell Counting Kit-8 (CCK-8) was purchased from Shanghai Tengyi Biotechnology Co., Ltd (Shanghai, China). Calcein/PI Cell Viability/Cytotoxicity Assay Kit, Annexin V-FITC Apoptosis Detection Kit and DAPI dihydrochloride (DAPI) were purchased from Shanghai Beyotime Biological Co., Ltd (Shanghai, China). Anti-BRD4 antibody was purchased from Abcam Plc. PD-L1/CD274 (C-terminal) Polyclonal antibody, HMGB1 Polyclonal antibody and calreticulin Polyclonal antibody were purchased from Proteintech Group, Inc (Wuhan, China). Anti-mouse PD-L1 antibody was obtained from BioXcell, Inc. CD3ε Rabbit mAb and CD8α Rabbit mAb were purchased from Cell Signaling Technology. Anti-mouse CD16/32, APC anti-mouse CD45, Brilliant Violet 421^TM^ anti-mouse CD8a, PE/Cyanine7 anti-mouse/human CD11b, APC anti-mouse CD11c, Brilliant Violet 421^TM^ anti-mouse FOXP3, PE anti-mouse CD25, PE/Cyanine7 anti-mouse/human CD3, Zombie Aqua^TM^ Dye were purchased from BioLegend.

**Instruments.** ^1^H-NMR and ^13^C-NMR spectra were recorded using 600 MHz NMR spectrometer (AVANCE Ⅲ HD 600, Bruker, German). High-resolution mass spectrometry analyses were performed on an LTQ Orbitrap Elite instrument (HRMS, Q Exactive Plus, thermoscientific, America). Ultraviolet-visible spectra and infrared spectrum of synthesized nanoparticles were obtained on a UV-vis absorption spectrophotometer (UV-1800, MAPADA, China) and fourier transform infrared spectrometer (FTIR; AVATAR 370, Nicolet, America). Size distribution and zeta potential were measured via dynamic light scattering (DLS; ZETASIZER PRO, Malvern, UK). The morphology was observed by transmission electron microscopy (TEM; JEM-F200, JEOL, Japan) and scanning electron microscope (SEM; GeminiSEM 300, ZEISS, German). The elemental analysis was carried out using X-ray photoelectron spectroscopy (XPS; K-Alpha, Thermo Scientific, America).

**Synthesis of JQ-COOH.** JQ-1 (500 mg , 1.09 mmol) was dissolved in 7 mL of dichloromethane (DCM). Trifluoroacetic acid (TFA) (2.5 mL, 33.15 mmol) was added to the solution, and the reaction mixture was stirred at room temperature for 3 hours. The solvent was then removed using a rotary evaporator. Isopropyl ether was added to the residue to precipitate the product, yielding a yellow solid.（Yield: 71.21%）。^1^H NMR (600 MHz, DMSO-d6) δ 7.50 (d, *J* = 8.2 Hz, 2H), 7.45 (d, *J* = 8.3 Hz, 2H), 4.47 (t, *J* = 7.1 Hz, 1H), 3.44 (dd, *J* = 16.7, 6.9 Hz, 1H), 3.33 (dd, *J* = 16.7, 7.4 Hz, 1H), 2.62 (s, 1H), 2.42 (s, 1H), 1.63 (s, 1H). ^13^C-NMR (151 MHz, DMSO-d6) ẟ 172.44, 163.72, 155.25, 150.53, 137.01, 135.81, 132.58, 131.42, 130.66, 130.37, 130.07, 128.98, 53.98, 36.93, 14.53, 13.16, 11.74. HRMS (ESI) m/z: Calcd. For C_19_H_17_ClN_4_O_2_S [M+H]^+^ 401.0761, found: [M+H]^+^ 401.0835.

**Synthesis of JQ-2C-TRT.** JQ-COOH (300 mg, 0.75 mmol), HATU (285 mg, 0.75 mmol), and DIPEA (392 μL, 0.25 mmol) were dissolved in 3.75 mL of DMF. 2-(tritylthio)ethanamine (240 mg, 0.75 mmol) and TEA (105 μL, 0.75 mmol) were added to this solution. The reaction mixture was stirred at room temperature overnight. After the reaction, an appropriate amount of ethyl acetate (EA) and an appropriate amount of water were added to the mixture. The layers were separated, and the organic phase was collected and concentrated to dryness using a rotary evaporator. The crude product was purified by column chromatography using a mixture of dichloromethane (DCM) and methanol (MeOH) (v/v, 30:1) as the eluent to afford a yellow solid.（Yield: 79.88%）。^1^H NMR (600 MHz, DMSO-d6) δ 8.32 (t, *J* = 5.6 Hz, 1H), 7.42 (d, *J* = 8.5 Hz, 2H), 7.35 – 7.31 (m, 14H), 7.25 (tt, *J* = 5.3, 3.2 Hz, 3H), 4.49 (dd, *J* = 8.3, 5.6 Hz, 1H), 3.24 (dd, *J* = 15.0, 8.6 Hz, 1H), 3.11 (dq, *J* = 12.9, 6.8, 6.3 Hz, 2H), 3.02 (dq, *J* = 13.1, 6.6 Hz, 1H), 2.61 (s, 3H), 2.41 (s, 3H), 2.28 (ddd, *J* = 11.7, 6.0, 5.0 Hz, 2H), 1.61 (s, 3H). ^13^C-NMR (151 MHz, DMSO-d6) ẟ 169.89, 163.65, 155.45, 150.49, 144.90, 136.97, 135.75, 132.60, 131.42, 130.68, 130.35, 130.10, 129.55, 128.80, 128.50, 127.22, 66.45, 54.17, 38.23, 37.91, 31.85, 14.53, 13.16, 11.75. HRMS (ESI) m/z: Calcd. For C_40_H_36_ClN_5_OS_2_ [M+H]^+^ 702.2050, [M+Na]^+^ 724.2050 found: [M+H]^+^ 702.2126, [M+Na]^+^ 724.1940.

**Synthesis of JQ-2C-SH.** JQ-2C-TRT (420 mg, 0.60 mmol) was dissolved in 5 mL of dichloromethane (DCM). Triisopropylsilane (TIPS) (300 μL, 1.29 mmol) and TFA (5 mL, 66.30 mmol) were added to this solution. The reaction mixture was stirred at room temperature for 2 hours. The solvent was then removed under reduced pressure using a rotary evaporator. The crude product was purified by column chromatography using a mixture of DCM and MeOH (v/v, 30:1) as the mobile phase to afford the final product as a yellow solid.（Yield: 71.60%）。^1^H-NMR (600 MHz, DMSO-d6) δ 8.41 (t, *J* = 5.6 Hz, 1H), 7.50 (d, *J* = 8.7 Hz, 2H), 7.44 (d, *J* = 8.4 Hz, 2H), 4.57 – 4.50 (m, 1H), 3.27 (ddd, *J* = 13.6, 10.4, 6.6 Hz, 5H), 2.61 (s, 2H), 2.57 (q, *J* = 7.4 Hz, 2H), 2.43 (d, *J* = 8.2 Hz, 1H), 2.42 (s, 3H), 1.63 (s, 3H). ^13^C-NMR (151 MHz, DMSO-d6) ẟ 170.05, 163.68, 155.48. 150.46, 137.08, 135.78, 132.65, 131.35, 130.68, 130.36, 130.08, 128.94, 54.23, 42.62, 38.00, 24.05, 14.53, 13.16, 11.76. HRMS (ESI) m/z: Calcd. For C_21_H_22_ClN_5_OS_2_ [M+H]^+^ 460.0954, [M+Na]^+^ 482.0954, found: [M+H]^+^ 460.1028, [M+Na]^+^ 482.0844.

**Synthesis of targeted-tumor peptide (C-PEG-RGD).** One gram of dichlororesin was weighed and activated with DCM. The first amino acid was coupled using N,N-Diisopropylethylamine (DIPEA) as the condensation system, and the reaction proceeded overnight at room temperature. After washing with DCM and DMF, the resin was capped with methanol for 30 minutes. The remaining amino acids and the PEG were coupled sequentially according to the sequence (C-PEG-RGD) using a 1-Hydroxybenzotriazole / N,N'-Diisopropylcarbodiimide (HOBT/DIC) condensation system for each coupling step. Each coupling reaction was carried out for 2 hours at room temperature. Deprotection of the Fmoc group was performed using a 20% piperidine solution in DMF. The crude peptide was cleaved from the resin using reagent K, and the final product was purified by HPLC. HRMS (ESI) m/z: Calcd. For C_22_H_40_N_8_O_10_S [M+H]^+^ 609.2588, [M+2H]^2+^ 305.1294, found: [M+H]^+^ 609.2657, [M+2H]^2+^ 305.1367.

**Preparation of PCN-CuS-JQ/RGD nanoparticles.** A mixture of 2248 mg of benzoic acid, 82 mg of tetrakis(4-Carboxyphenyl)Porphyrin (TCPP) and 202 mg of iron(III) chloride hexahydrate was dissolved in 50 mL of DMF. The reaction proceeded with stirring at 90 °C for 5 hours. The product was then collected by centrifugation at 12,000 rpm for 15 minutes and washed with DMF to remove unreacted starting materials, yielding the metal-organic framework PCN(Fe). In a separate preparation, 80 mg of PVP(K30) and 14 mg of copper(II) chloride dihydrate (CuCl_2_·2H_2_O) were dissolved in 80 mL of deionized water with stirring at room temperature for 30 minutes. Then, 0.32 mL of an aqueous sodium sulfide nonahydrate(Na_2_S·9H_2_O) solution (60.54 mg/mL) was added, and the mixture was stirred for 5 minutes. This mixture was then heated to 98 °C and stirred for 1 hour. After cooling to room temperature, 2 mL of a PCN(Fe) dispersion (20 mg/mL, 40 mg) was added dropwise to the solution, followed by stirring at room temperature for 1 hour. The product (PCN-CuS) was isolated by centrifugation at 12,000 rpm for 15 minutes and washed with deionized water. Finally, 10 mg of PCN-CuS and 10 mg of C-PEG-RGD were dissolved in 7.5 mL of deionized water and stirred at room temperature for 3 hours. Separately, 10 mg of JQ-2C-SH was dissolved in 2.5 mL of methanol and then added to the mixture. The resulting mixture was stirred overnight at room temperature. The final product (PCN-CuS-JQ/RGD) was collected by centrifugation at 12,000 rpm for 15 minutes and washed with deionized water.

**Hemolysis experiment.** To evaluate the hemocompatibility of PCN-CuS-JQ/RGD, different concentrations of the nanosystem were prepared and dispersed in PBS. Then 1 mL of each nanosystem solution, 1 mL of ultrapure water (positive control) and 1 mL of PBS solution (negative control) were mixed with 300 μL of red blood cells (RBCs), respectively. The mixtures were incubated at 37 °C for 4 hours. After incubation, they were centrifuged at 3000 rpm for 15 minutes. Subsequently, 100 μL of the supernatant from each sample was transferred to a 96-well plate and the absorbance at 542 nm was measured. The hemolysis rate was calculated based on the absorbance values.

**Photothermal effect and** **photothermal conversion efficiency.** To investigate the photothermal effect of PCN-CuS-JQ/RGD, aqueous solutions of the nanosystem at different concentrations (0, 25, 50, 75 and 100 μg/mL) were prepared. These solutions were then irradiated with a 1064 nm laser (1.02 W/cm²), and the temperature change of each solution was monitored every minute for 5 minutes. Additionally, the nanosystem (50 μg/mL) was prepared and irradiated with 1064 nm laser at different power densities (0.25, 0.51, 1.02 and 1.53 W/cm²). The temperature change of the solution was monitored every minute for 5 minutes under each condition. To evaluate the photothermal stability of the nanosystem, the PCN-CuS-JQ/RGD aqueous solution (50 μg/mL) was subjected to five on/off cycles of laser irradiation. In each cycle, the solution was irradiated with the 1064 nm laser (1.02 W/cm²) for 5 minutes, followed by turning off the laser and allowing the solution to cool down to room temperature. The temperature was recorded every 15 seconds during the entire process. The photothermal conversion efficiency of the PCN-CuS-JQ/RGD solution (50 μg/mL) was determined based on a temperature increase of 17.2 °C achieved within 5 minutes under 1064 nm laser irradiation.

Calculation of light-to-heat conversion efficiency:

The photothermal conversion efficiency (η) was measured according to the previously described method.

η=ℎ𝑠(∆𝑇_material_−∆𝑇_water_)/[𝐼(1−10^−𝐴^)] —— formula 1

∆𝑇_material_ and ∆𝑇_water_ are the temperature changes of the test sample and the blank sample (H_2_O), respectively. ℎ is the heat transfer coefficient. 𝑠 is the surface area of the container. 𝐼 is the laser power density. *A* is the absorbance at 1064 nm. And the value of ℎ𝑠 is determined by formula 2.

ℎ𝑠 =𝑚𝐶/𝜏_𝑠_ —— formula 2

𝑚 is the mass of the solution (about 0.25 g). 𝐶 is the specific heat capacity of the solution (H_2_O is 4.2 J/(g·℃)), 𝜏_𝑠_ is the related time constant, which can be determined by formula 3 during the cooling cycle.

t=−𝜏_𝑠_ln (𝜃) —— formula 3

𝜃 is the dimensionless parameter that changes over time, called the driving force temperature, defined as formula 4.

𝜃 =(𝑇−𝑇_𝑠𝑢𝑟𝑟_)/(𝑇_𝑚𝑎𝑥_−𝑇_𝑠𝑢𝑟𝑟_) —— formula 4

𝑇_𝑚𝑎𝑥_ and 𝑇_𝑠𝑢𝑟𝑟_ are the maximum steady state temperature and the environmental temperature.

**Decomposition of** **PCN-CuS-JQ/RGD under GSH.** To verify the decomposition of PCN-CuS-JQ/RGD in the tumor microenvironment, the nanosystem (2 mg/mL) was dispersed in H₂O and 5 mM GSH solution respectively. The mixtures were then shaken at room temperature. These Samples were collected at 0 hours and 48 hours for TEM specimen preparation, and their morphologies were observed by transmission electron microscopy (TEM).

**Cell culture.** 4T1 cells were cultured in Dulbecco’s Modified Eagle Medium (DMEM) containing 10 % fetal bovine serum (FBS) and 1 % penicillin-streptomycin (PS). The culture condition was 37 ℃ incubator with 5 % CO_2_.

***In vitro* cell uptake and colocalization of** **PCN-CuS-JQ/RGD.** PCN-CuS-JQ/RGD was mixed with Rhodamine B at a 1:1 mass ratio in water and stirred overnight at room temperature to load the Rhodamine B onto the nanosystem. Fluorescence lifetime imaging microscopy (Leica, STELLARIS 8; German) was used to characterize the intracellular distribution of the nanoparticles in 4T1 cells. 4T1 cells were seeded in confocal dishes at a density of 1×10⁵ cells per well and incubated for 36 hours. To investigate the effect of incubation time and concentration on cellular uptake, the cells were treated as follows: (1) Concentration-dependent uptake: Treated with 12.5, 25 and 50 μg/mL of the PCN-CuS-JQ/RGD + Rhodamine B complex for 4 hours. (2) Time-dependent uptake: Treated with 50 μg/mL of the complex for 1, 2 and 4 hours. After the respective treatments, the cells were washed with PBS, fixed with 4% paraformaldehyde (PFA) for 15 minutes, and washed again with PBS. Cell nuclei were then stained with DAPI at 37°C for 30 minutes. Finally, the cells were imaged in PBS.

**CCK8 assay.** 4T1 cells were seeded in a 96-well plate at a density of 2,500 cells per well and cultured for 24 hours. The cells were then treated with PCN-CuS-JQ/RGD at concentrations of 0, 25, 50, and 100 μg/mL. As a control, cells were also treated with the same concentrations of PCN-CuS-RGD. For each concentration, both laser-treated and non-laser-treated groups were established. After 24 hours of treatment, the laser-treated groups were irradiated with a 1064 nm laser at a power density of 2.04 W/cm² for 5 minutes. Following an additional 24 hours of incubation post-laser irradiation, CCK-8 reagent was added to each well. The absorbance of each well was measured at a wavelength of 450 nm.

**Live/dead cell staining assay.** 4T1 cells cultured in confocal dishes were divided into 6 groups (PBS, PBS+L, PCN-CuS-RGD, PCN-CuS-RGD+L, PCN-CuS-JQ/RGD, PCN-CuS-JQ/RGD+L). Cells in each group were incubated with the respective materials at a concentration of 50 μg/mL. Following incubation, the groups were irradiated with a 1064 nm laser at a power density of 2.04 W/cm² for 5 minutes, while the non-laser groups were not irradiated. 24 hours after the laser treatment, all cells were washed with PBS. Subsequently, 300 μL of a Calcein-AM/PI staining working solution was added to each dish, and the cells were incubated at 37°C for 30 minutes. Finally, the cells were subjected to confocal fluorescence imaging.

**Flow cytometry analysis of apoptosis.** 4T1 cells were cultured in 6-well plates and randomly divided into 6 groups (PBS, PBS+L, PCN-CuS-RGD, PCN-CuS-RGD+L, PCN-CuS-JQ/RGD, PCN-CuS-JQ/RGD+L). Cells in each group were incubated with the respective materials at a concentration of 50 μg/mL. The laser groups were then irradiated with a 1064 nm laser at a power density of 2.04 W/cm² for 5 minutes, while the non-laser groups were not treated. 24 hours after laser treatment, both the cell culture supernatant and the adherent cells from each well were collected. The cells were stained with FITC-Annexin V and Propidium Iodide (PI) in the dark for 20 minutes. Apoptosis was subsequently analyzed using flow cytometry.

**Western blotting experiment.** 4T1 cells were cultured in 6-well plates to generate protein samples from 6 experimental groups: PBS, PBS+L, PCN-CuS-RGD, PCN-CuS-RGD+L, PCN-CuS-JQ/RGD and PCN-CuS-JQ/RGD+L. Cell treatment was identical to the previously described method, except the incubation concentration of the materials was adjusted to 25 μg/mL. The cells were lysed using Cell Lysis Buffer for Western and IP. The protein concentration was determined using the BCA protein assay kit. Subsequently, loading buffer was added to the protein samples, which were then denatured by heating at 100°C for 10 minutes using a metal bath. The extracted proteins were separated by 10% sodium dodecyl sulfate polyacrylamide gel electrophoresis (SDS-PAGE). The separated proteins, including the target proteins and internal reference proteins, were then transferred onto a solid support membrane (PVDF/NC). The membrane was blocked with 5% skim milk for 1 hour, followed by incubation with specific primary antibodies at 4°C overnight. After washing, the membrane was incubated with corresponding secondary antibodies at room temperature for 1 hour. Finally, the protein bands were visualized by adding an ECL substrate and imaging the membrane using a gel imaging system for Western blot analysis.

***In vitro* ICD evaluation.** The cell treatment was consistent with the method described above. The treated 4T1 cells were stained with anti-CRT antibody (Cy3-conjugated rabbit-anti-goat IgG) and anti-HMGB 1 antibody (Cy3-conjugated rabbit-anti-goat IgG), respectively. Cell nuclei were counterstained with DAPI in the dark at room temperature. Finally, the immunofluorescence in each group was detected and imaged using a laser scanning confocal microscope.

**The establishment of mouse tumor model.** Balb/C mice (6-7 weeks old, female, 16-18 g) were procured from a supplier at Huachuan Xinnuo Pharmaceutical Technology Co., Ltd in Jiangsu, China. Animal experiments were reviewed and approved by the Ethics Committee of Shanghai University (approval number ECSHU 2024-118). 4T1 tumor model was established in female Balb/C mice by subcutaneously injecting 10^6^ 4T1 cells into the right hip of each mouse. When the tumor volume reached 75-100 mm³, the mice were randomly assigned to different experimental groups.

***In vivo* biodistribution.** PCN-CuS-JQ/RGD was mixed with indocyanine green (ICG) at a 1:1 mass ratio in methanol and stirred overnight to load the ICG onto the nanosystem. 4T1 tumor-bearing mice were intravenously injected via the tail vein with either free ICG or PCN-CuS-JQ/RGD@ICG at a dose of 10 mg/kg (ensuring an equivalent ICG dose between groups). The fluorescence signals of ICG in the mice were then monitored at 2, 4, 6, 8, 10, 12, 24, 36 and 48 hours post-injection using an in vivo imaging system (IVIS Spectrum; PerkinElmer, USA). At the end of the study, all mice were euthanized, and the major organs and tumors were harvested for *ex vivo* fluorescence imaging to analyze the biodistribution of the nanosystem.

***In vivo*** ***T*_1_-weighted MRI.** 4T1 tumor-bearing Balb/C mice were intravenously injected via the tail vein with PBS, ferric chloride, or a PCN-CuS-JQ/RGD solution. *T*_1_-weighted magnetic resonance imaging (MRI) was performed at different time points (0, 2 and 4 hours) post-injection. Additionally, *T*_1_-weighted MRI was conducted on the PCN-CuS-JQ/RGD solutions at different concentrations (0.625, 1.25, 2.5, 5 and 10 mg/mL).

**Living tumor thermal imaging.** To monitor the temperature changes in vivo under laser irradiation, when the tumor volume reached approximately 100 mm³, two mice were intravenously injected with PCN-CuS-JQ/RGD (10 mg/kg) and PBS, respectively. Eight hours post-injection, the mice were anesthetized, and the tumor regions were irradiated with a 1064 nm laser (1.02 W/cm²) for 5 minutes. During the irradiation process, the temperature changes at the tumor site were continuously monitored, and thermal imaging pictures were captured every minute.

***In vivo* antitumor efficiency.** Tumor-bearing mice were randomly divided into five groups:

(1) PBS, (2) PCN-CuS-RGD, (3) PCN-CuS-RGD+L, (4) PCN-CuS-JQ/RGD, (5) PCN-CuS-JQ/RGD+L. Mice in each group received intravenous injections of the respective formulations at a dose of 10 mg/kg every three days, for a total of five injections. Twenty-four hours after each injection, the tumor area of mice in the laser-treated groups was irradiated with a 1064 nm laser at a power density of 1.02 W/cm² for 5 minutes. Mouse body weight was measured, and tumor volume was monitored with a caliper every three days. Tumor volume was calculated using the formula: Volume = π/6×L×W^2^, where L is the length and W is the width. After the treatment regimen, all mice were euthanized. The harvested tumors were fixed in formalin, embedded in paraffin, and sectioned. The sections were then subjected to Hematoxylin and Eosin (H&E) staining. Additionally, immunohistochemical and immunofluorescence staining were performed on the tumor sections to detect the expression levels of various target indicators.

***In vivo* biological safety experiment.** To evaluate the potential toxicity of the different formulations, major organs (heart, liver, spleen, lungs, and kidneys) and blood samples were collected from mice in each group at the end of the treatment period. The organ tissues were subjected to hematoxylin and eosin (H&E) staining. Blood samples were used for complete blood count analysis and blood biochemical tests.

**Flow cytometry analysis.** The tumor tissues were minced and digested in DMEM medium containing collagenase and DNase at 37°C with agitation for 1 hour to obtain a single-cell suspension. Lymph nodes were ground to prepare a cell suspension. The cell suspensions were then centrifuged at 2500 rpm for 5 minutes to pellet the cells, and red blood cells were removed using a red blood cell lysis solution. Subsequently, the cells were stained with a series of specific antibodies. Finally, the stained cells were analyzed using the flow cytometer. All data processing was performed using FlowJo software. The gating strategy for each cell population is as follows: mature dendritic cells (CD11b^+^ → CD11c^+^CD80^+^); Tregs (CD45⁺ → CD3⁺ → CD4⁺ → CD25⁺Foxp3⁺); NK cells (CD45^+^ → CD3^-^ → CD49b^+^); CD4^+^ T cells (CD45⁺ → CD3⁺ → CD4⁺); CD8a^+^ T cells (CD45⁺ → CD3⁺ → CD8a⁺).

**Secondary tumor study.** A bilateral tumor model was established in Balb/C mice by injecting 1×10^6^ 4T1 cells into the right flank, followed by inoculation of 5×10^5^ 4T1 cells into the left flank four days later. When the volume of the primary (right) tumor reached approximately 100 mm³, the mice were randomly divided into four groups: (1) PBS, (2) aPD-L1, (3) PCN-CuS-JQ/RGD+L, (4) aPD-L1+PCN-CuS-JQ/RGD+L. The treatment was administered in cycles, with one cycle every three days for a total of four cycles. aPD-L1 was administered intraperitoneally at a dose of 2.5 mg/kg on days 0 and 3. PCN-CuS-JQ/RGD was administered intravenously on days 0, 3, 6 and 9. For the laser-treated groups, irradiation was performed 24 hours after each nanosystem injection. Tumor volumes on both sides were recorded every three days. Upon completion of the treatment, all tumors were excised, weighed, and photographed.

**Image source attribution.** Scheme 1, Figure 6A and Figure 8A were created using BioRender (BioRender.com).

**Statistical analysis.** Statistical analysis was conducted by Student’s t test for comparison of two groups and one-way ANOVA for multiple groups.

**Supporting figures**

**
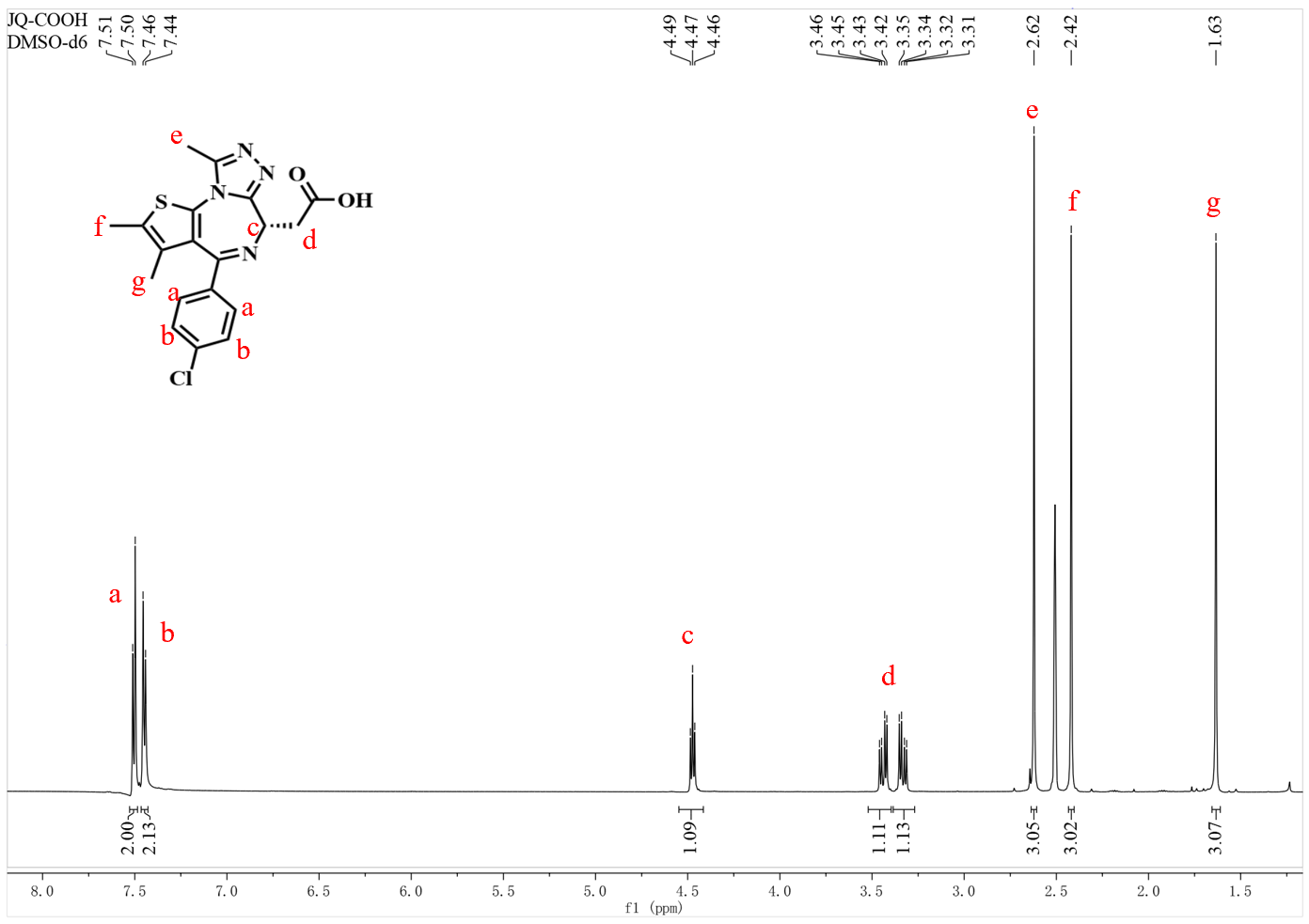
**

**Figure S1.** ^1^H-NMR (600 MHz, DMSO-d6) spectrum of JQ-COOH.


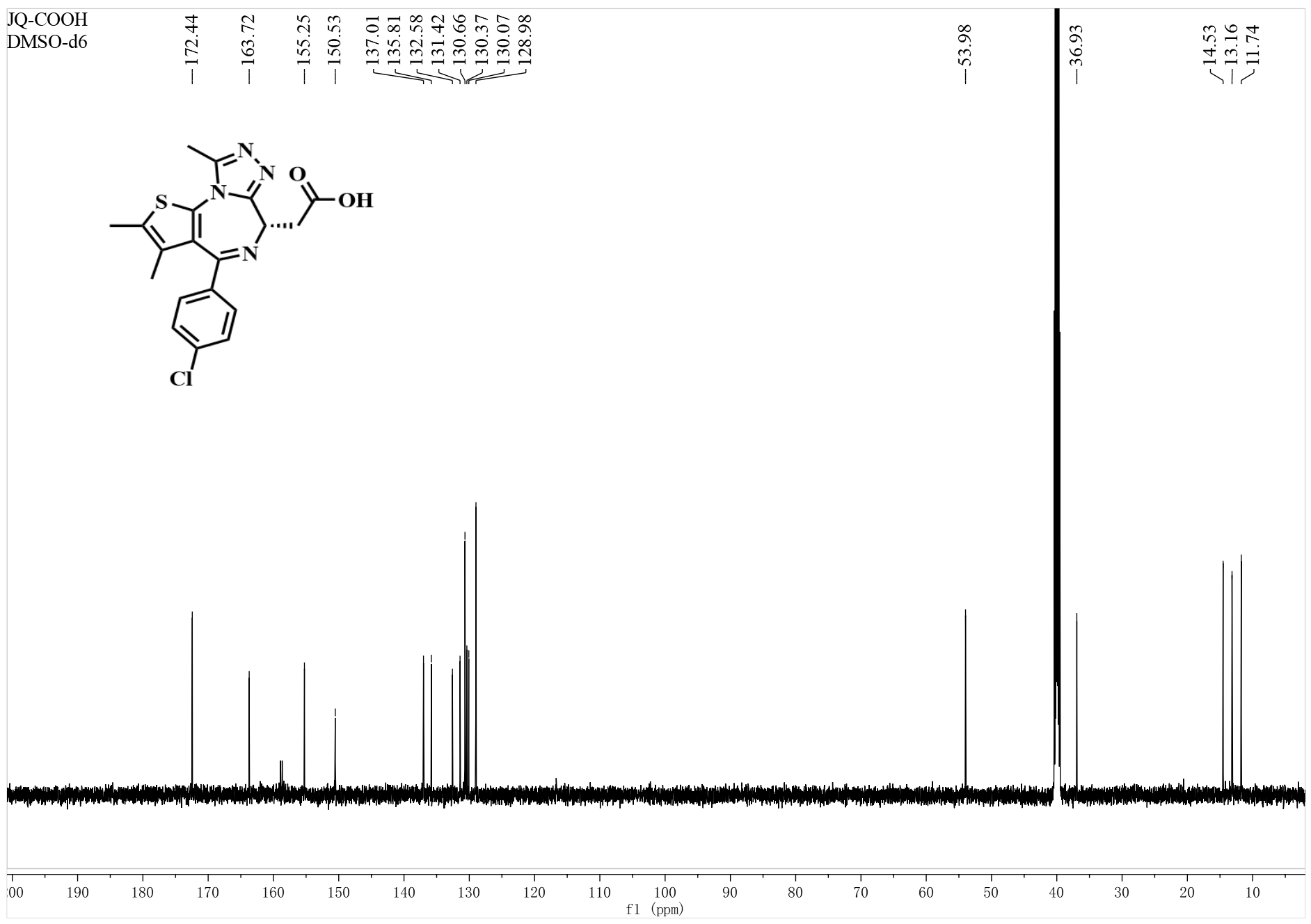


**Figure S2.** ^13^C-NMR (151 MHz, DMSO-d6) spectrum of JQ-COOH.


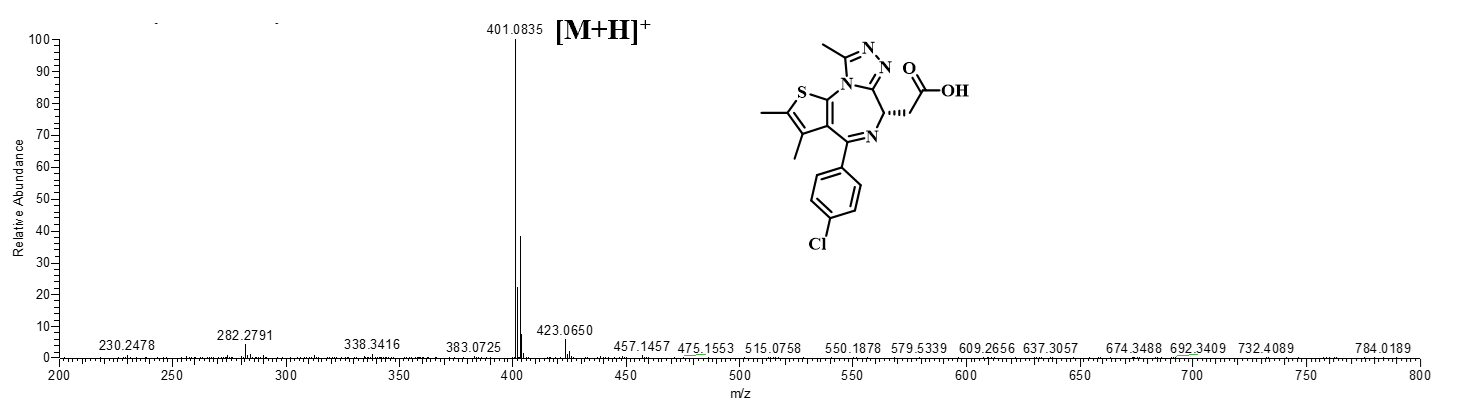


**Figure S3.** HRMS spectrum of JQ-COOH.


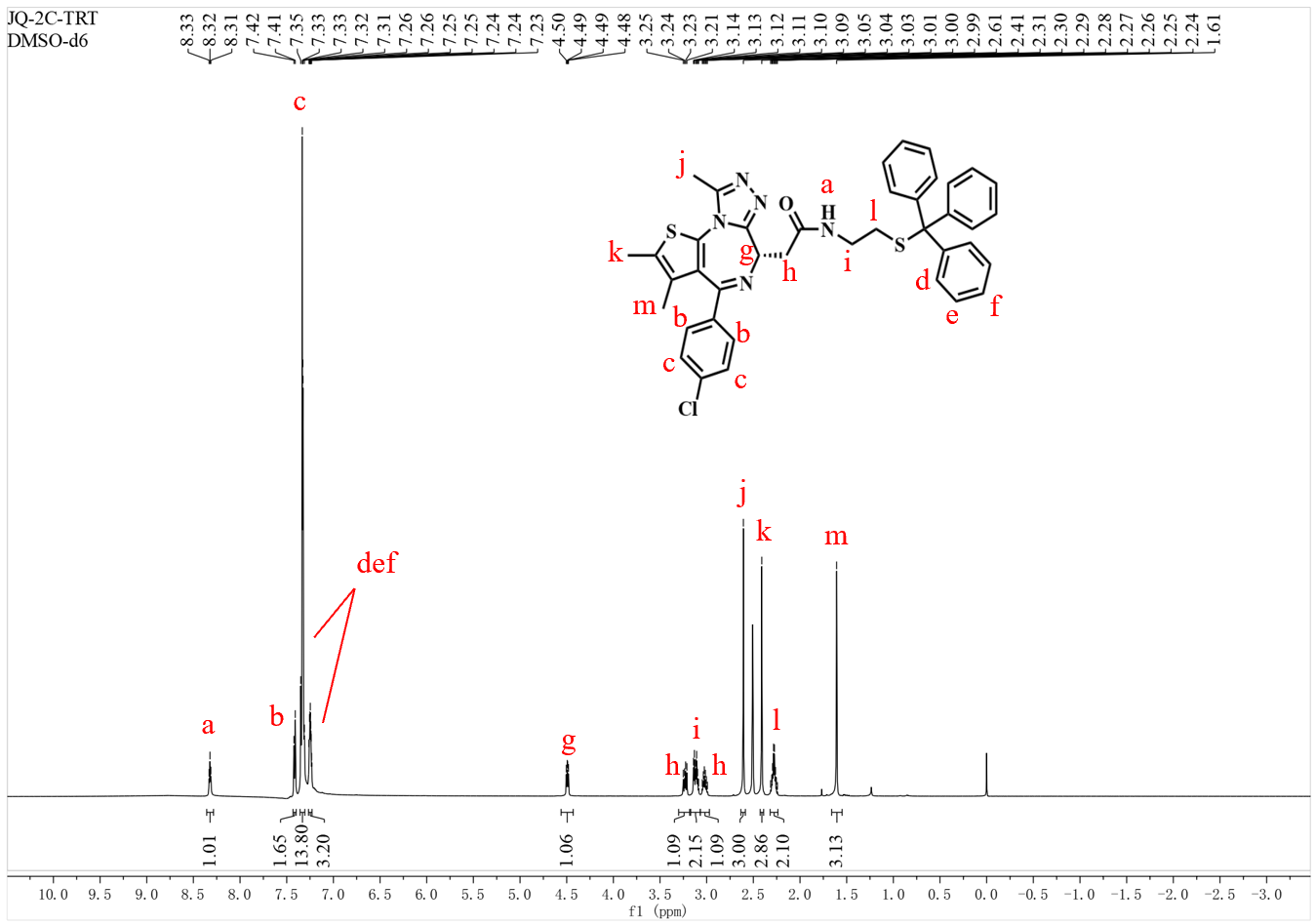


**Figure S4.** ^1^H-NMR (600 MHz, DMSO-d6) spectrum of JQ-2C-TRT.


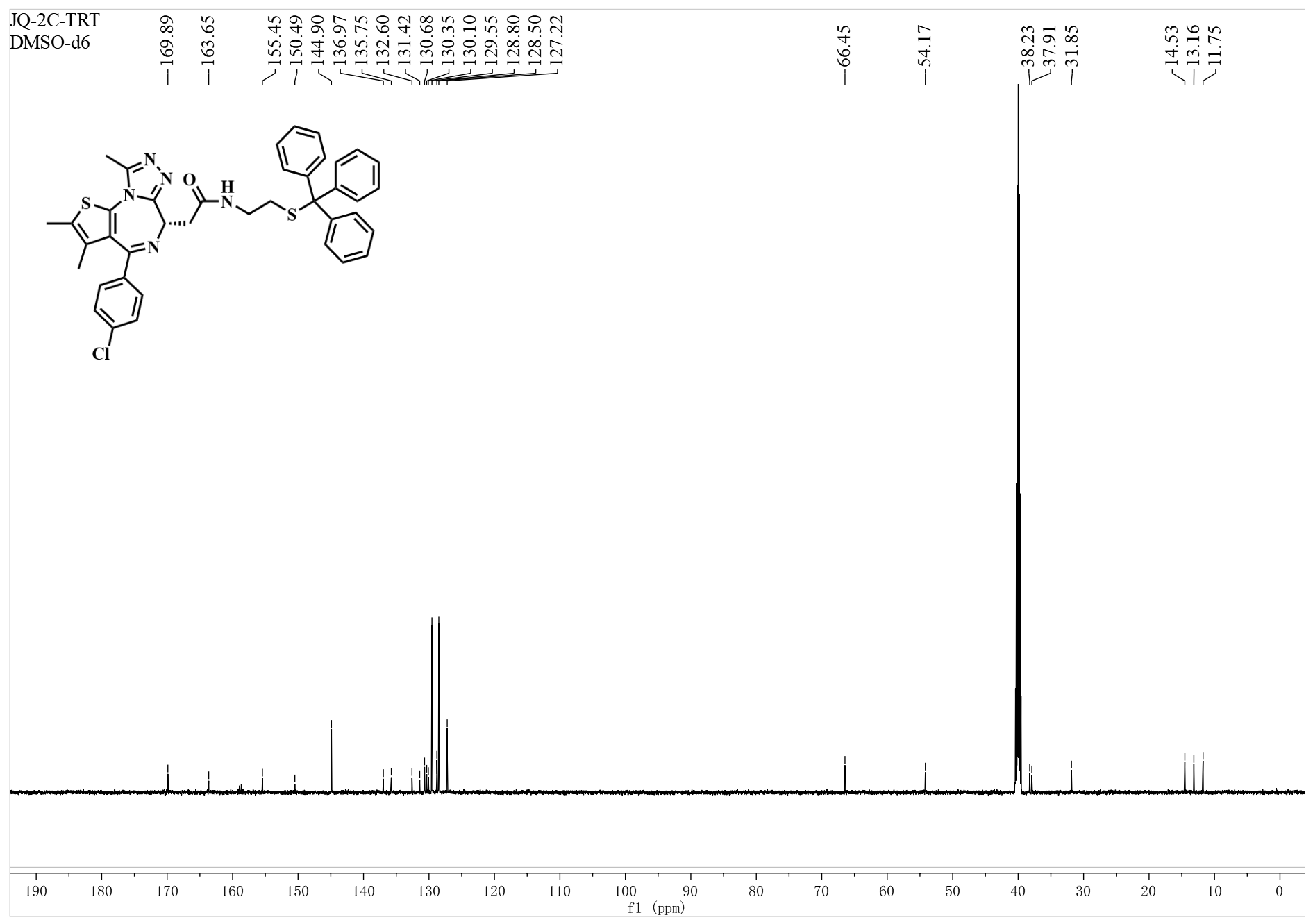


**Figure S5.** ^13^C-NMR (151 MHz, DMSO-d6) spectrum of JQ-2C-TRT.


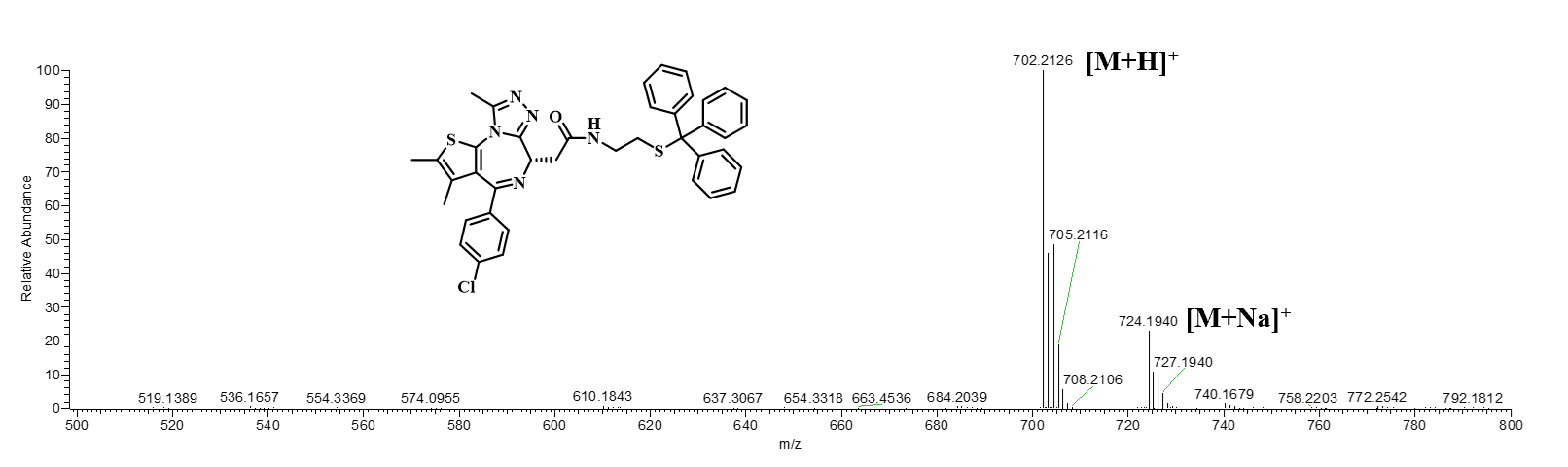


**Figure S6.** HRMS spectrum of JQ-2C-TRT.


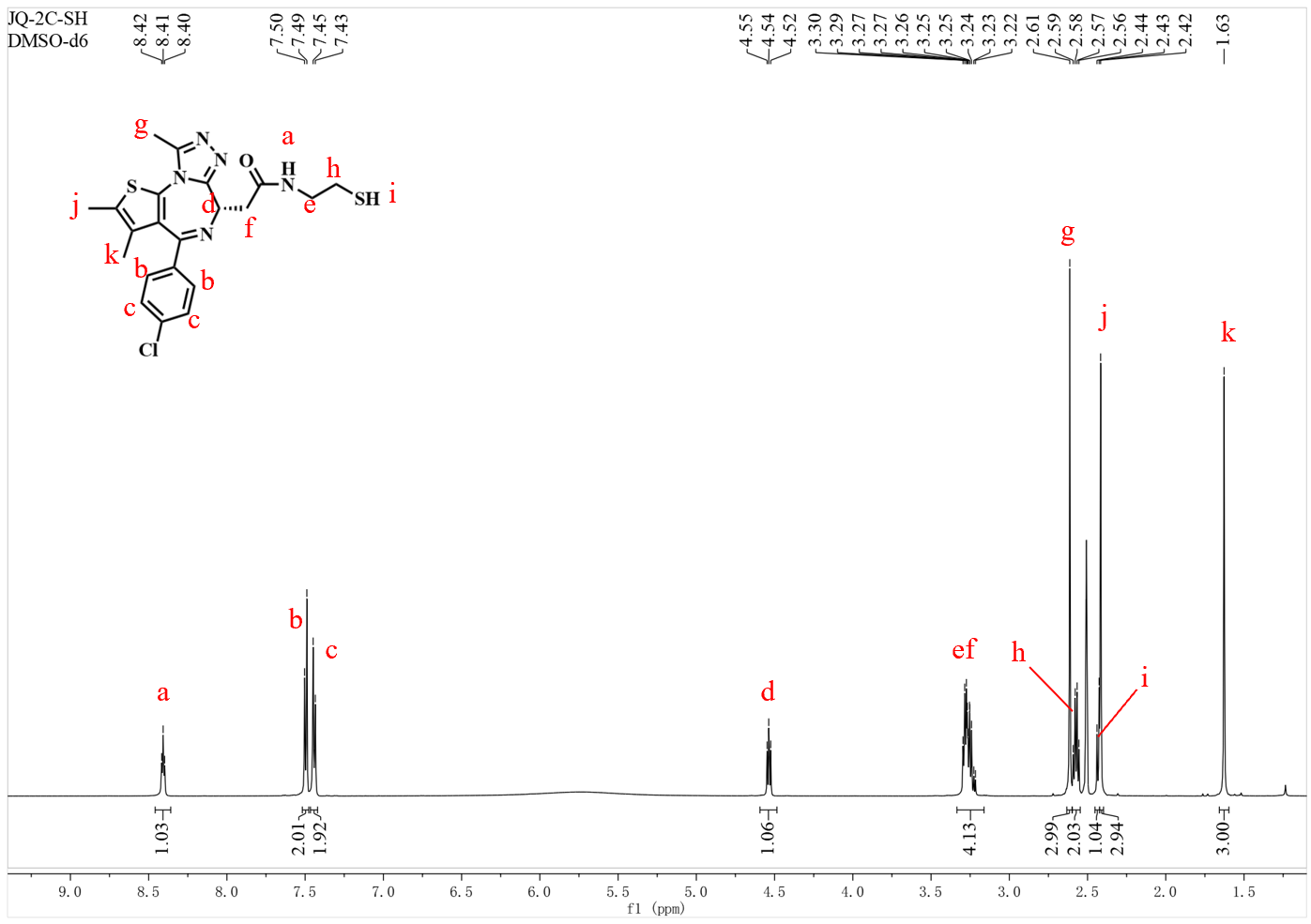


**Figure S7.** ^1^H-NMR (600 MHz, DMSO-d6) spectrum of JQ-2C-SH.


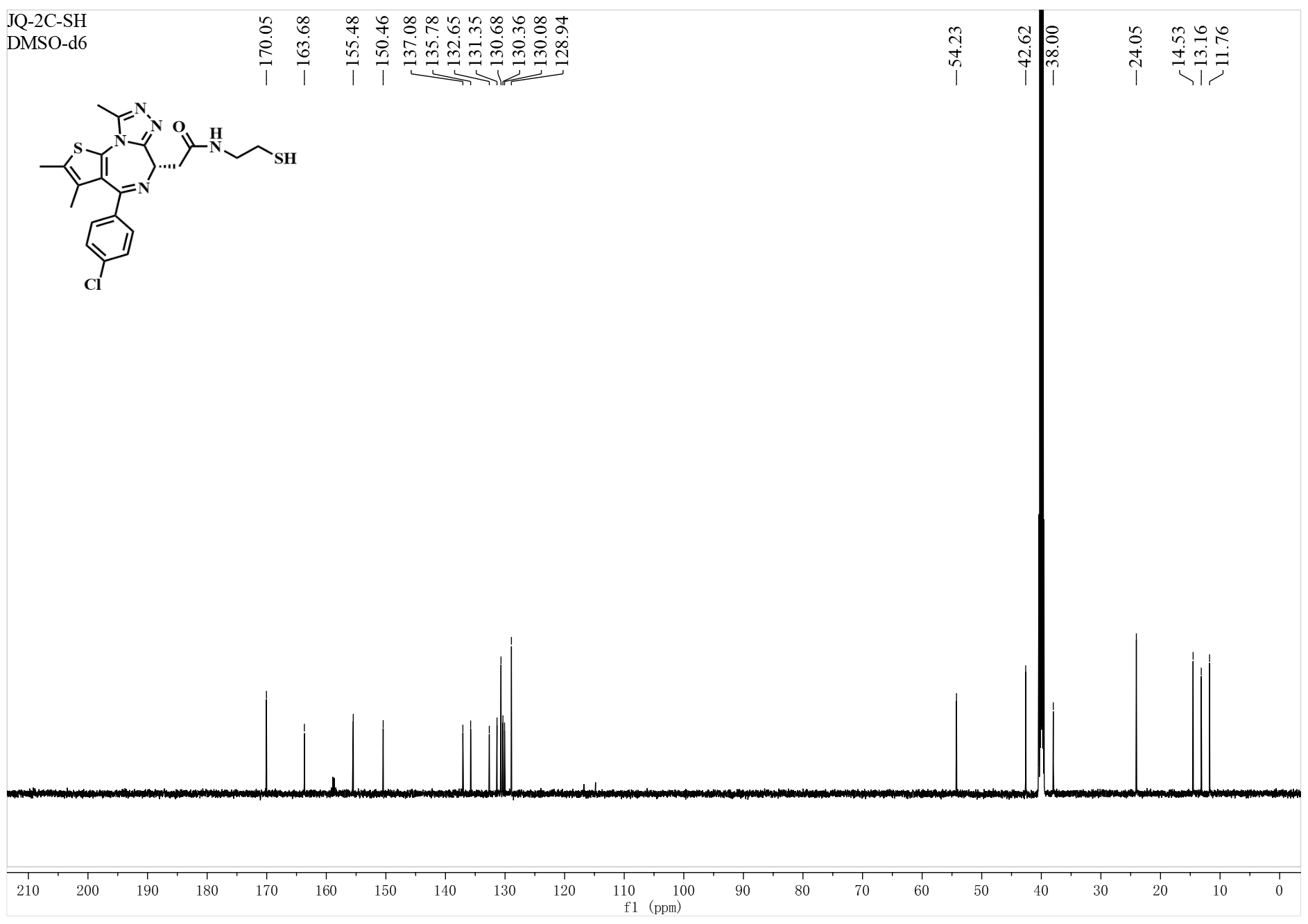


**Figure S8.** ^13^C-NMR (151 MHz, DMSO-d6) spectrum of JQ-2C-SH.


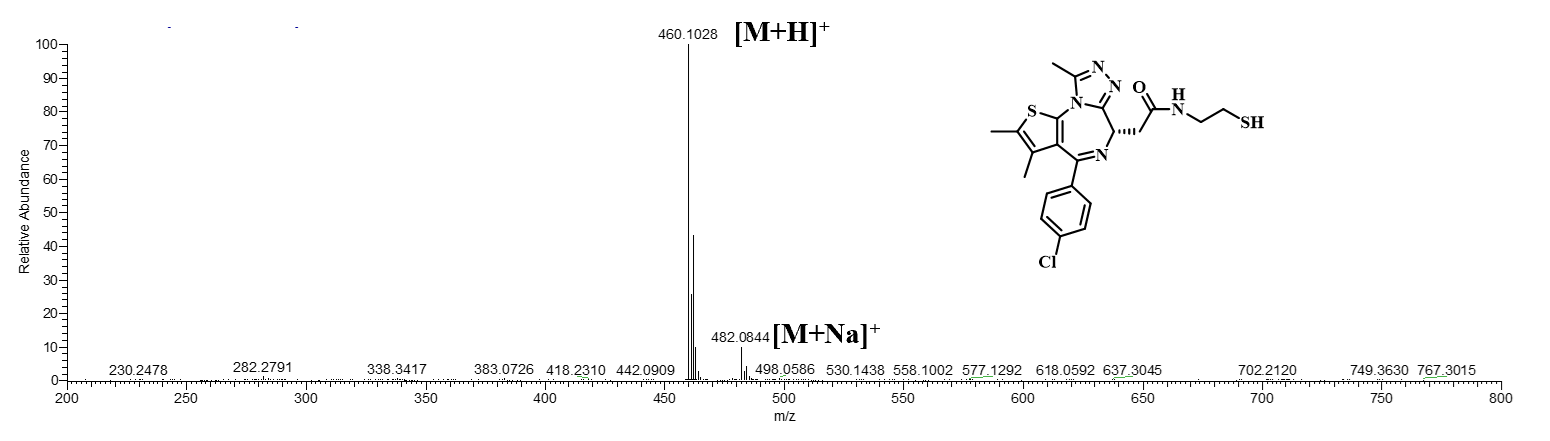


**Figure S9.** HRMS spectrum of JQ-2C-SH.





**Figure S10.** The HPLC diagram of C-PEG-RGD at 214 nm.

**Table S1.** Purity of C-PEG-RGD

| Peak No. | Ret time | Height | Area | Concentration |
| --- | --- | --- | --- | --- |
| 1 | 10.662 | 386821 | 8059750 | 96.350 |
| 2 | 11.820 | 14491 | 305315 | 3.650 |
| Total |  | 401311 | 8365065 |  |


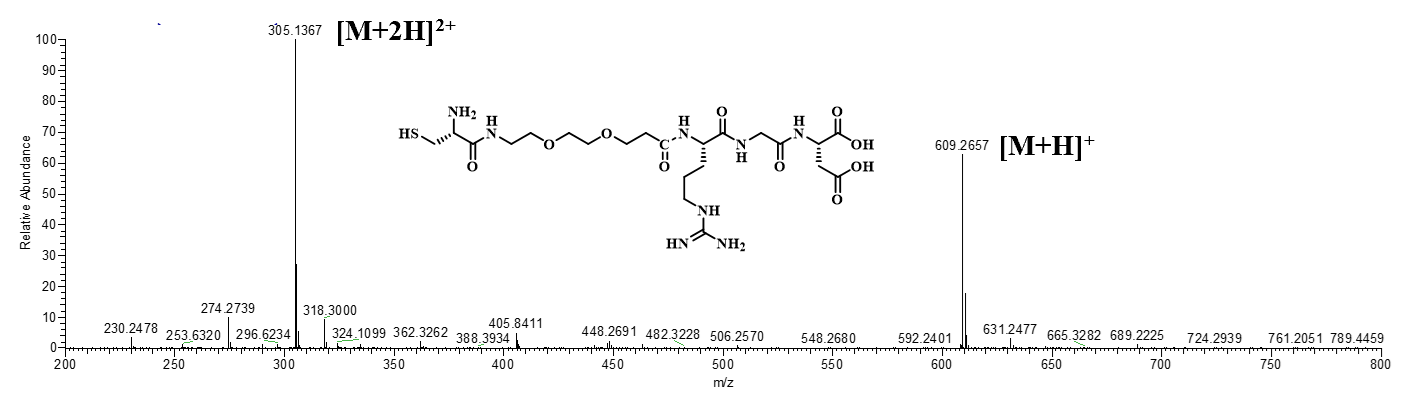


**Figure S11.** HRMS spectrum of C-PEG-RGD.





**Figure S12.** High-resolution Cl 2p XPS spectrum in PCN-CuS-JQ/RGD.





**Figure S13.** The XRD pattern of PCN(Fe) and PCN-CuS.


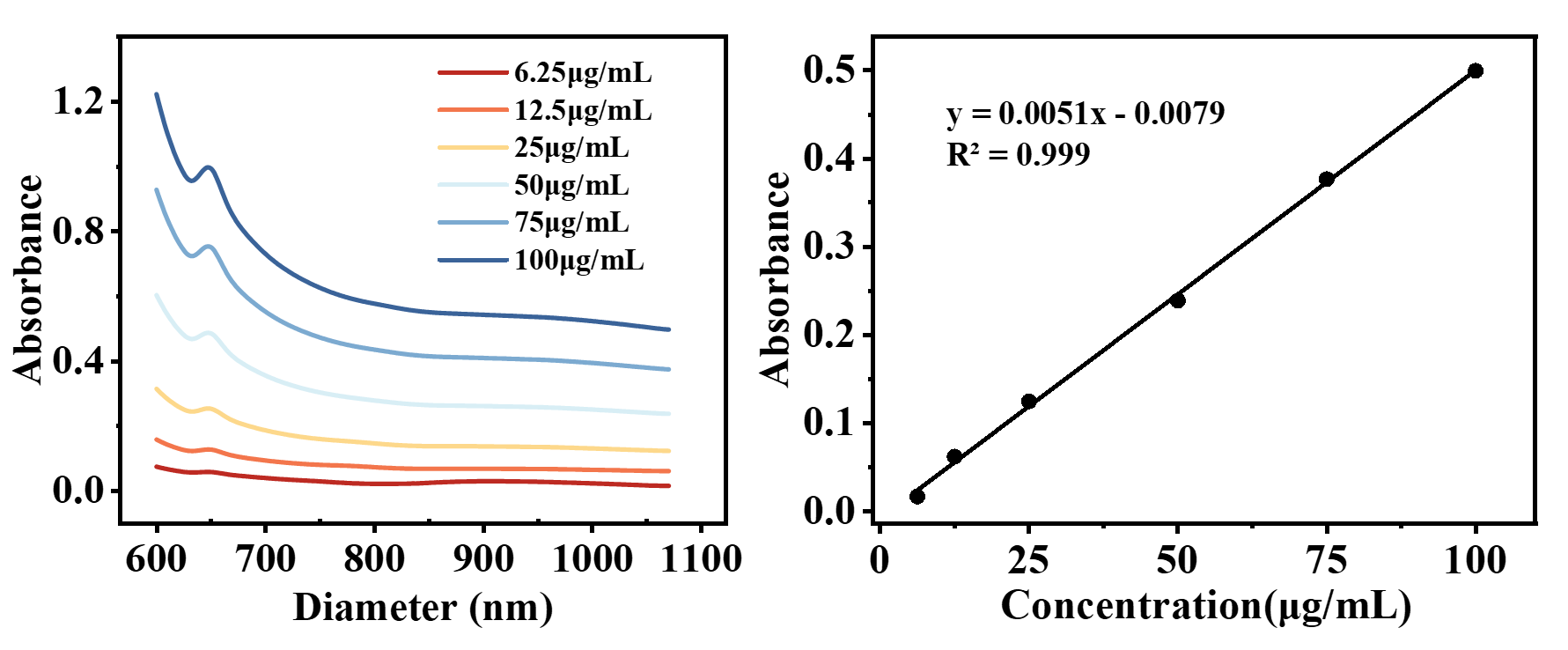


**Figure S14.** UV-Vis absorption spectra of PCN-CuS-JQ/RGD.


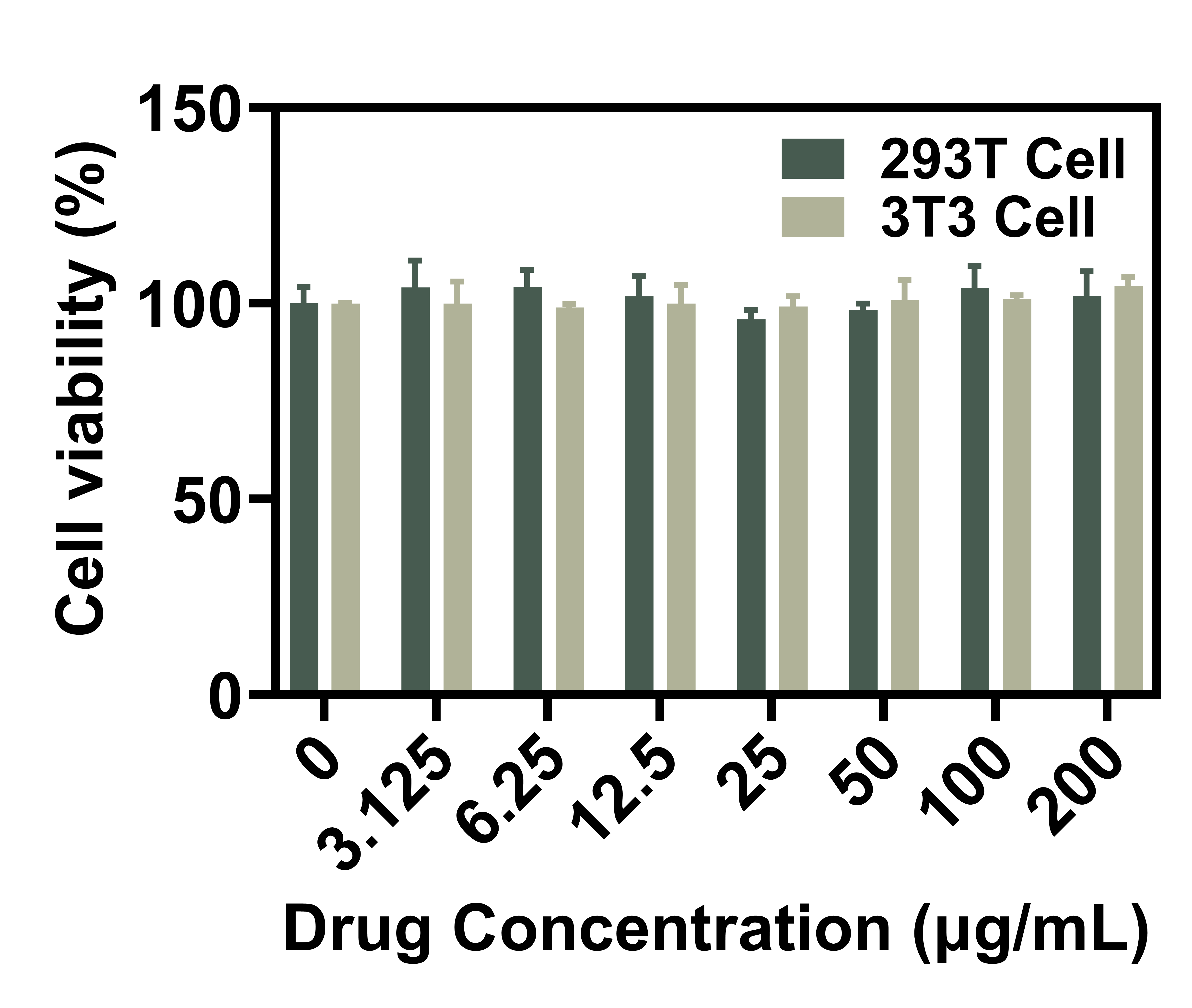


**Figure S15.** Cytotoxicity of PCN-CuS-JQ/RGD against 293T and 3T3 cells assessed by CCK8. Data are presented as mean ±  s.e.m. (n=3).


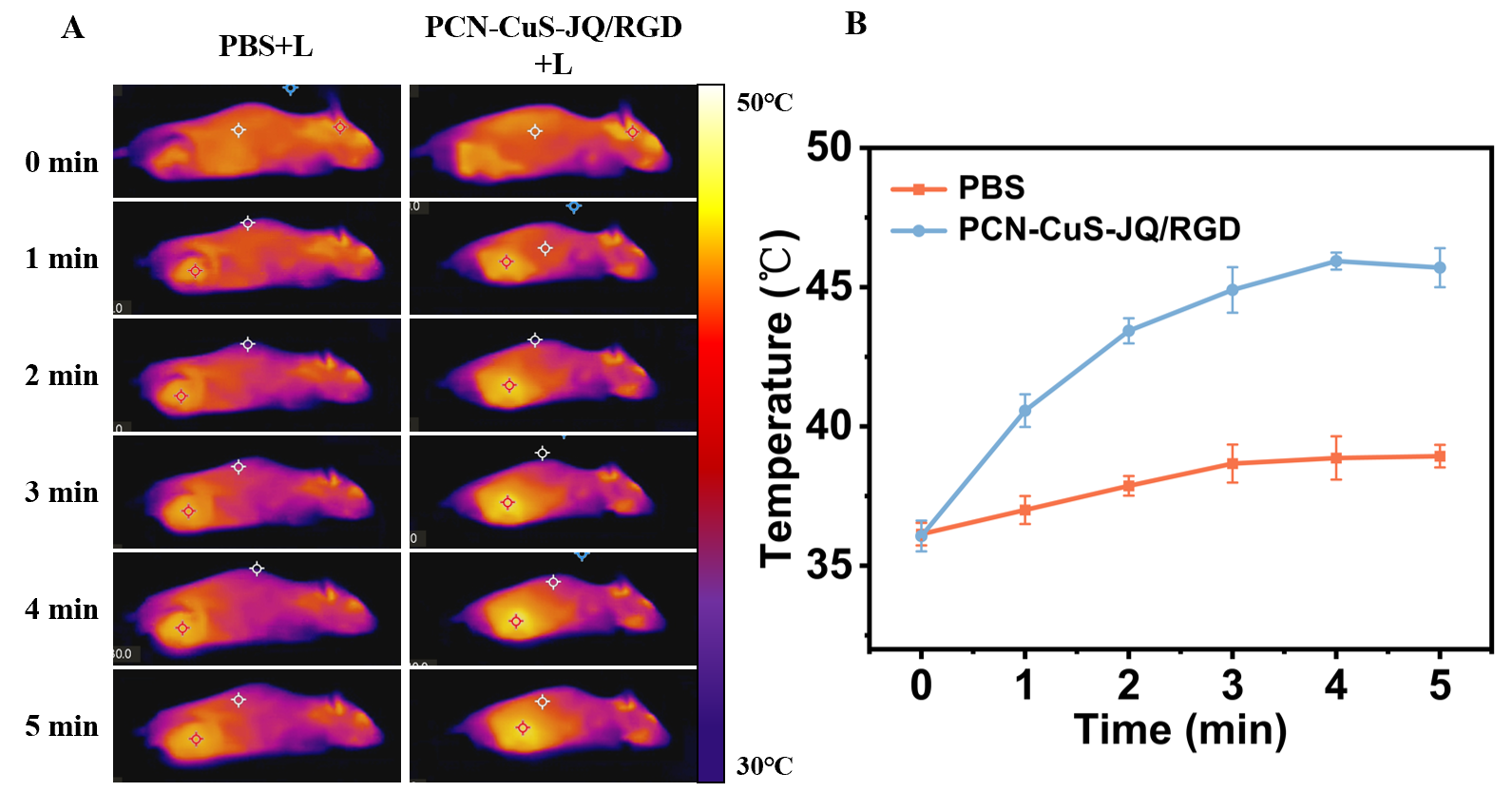


**Figure S16.** Thermal images (A) and temperature curve (B) of 4T1 tumor-bearing mice after injection of PBS or PCN-CuS-JQ/RGD under laser irradiation for 5 min. Data are presented as mean ±  s.e.m. (n=3).


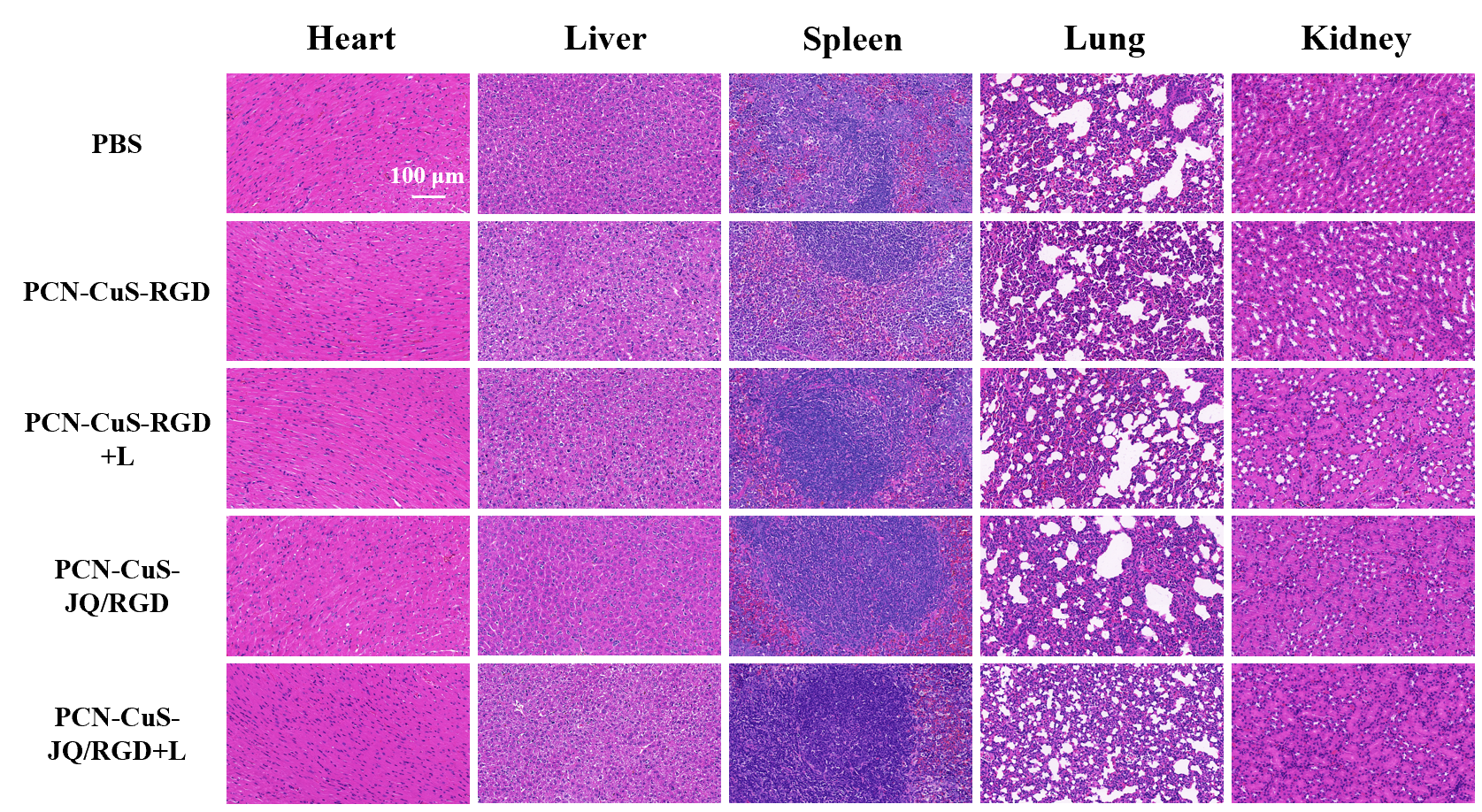


**Figure S17.** H&E staining of major organs from mice after different treatments.


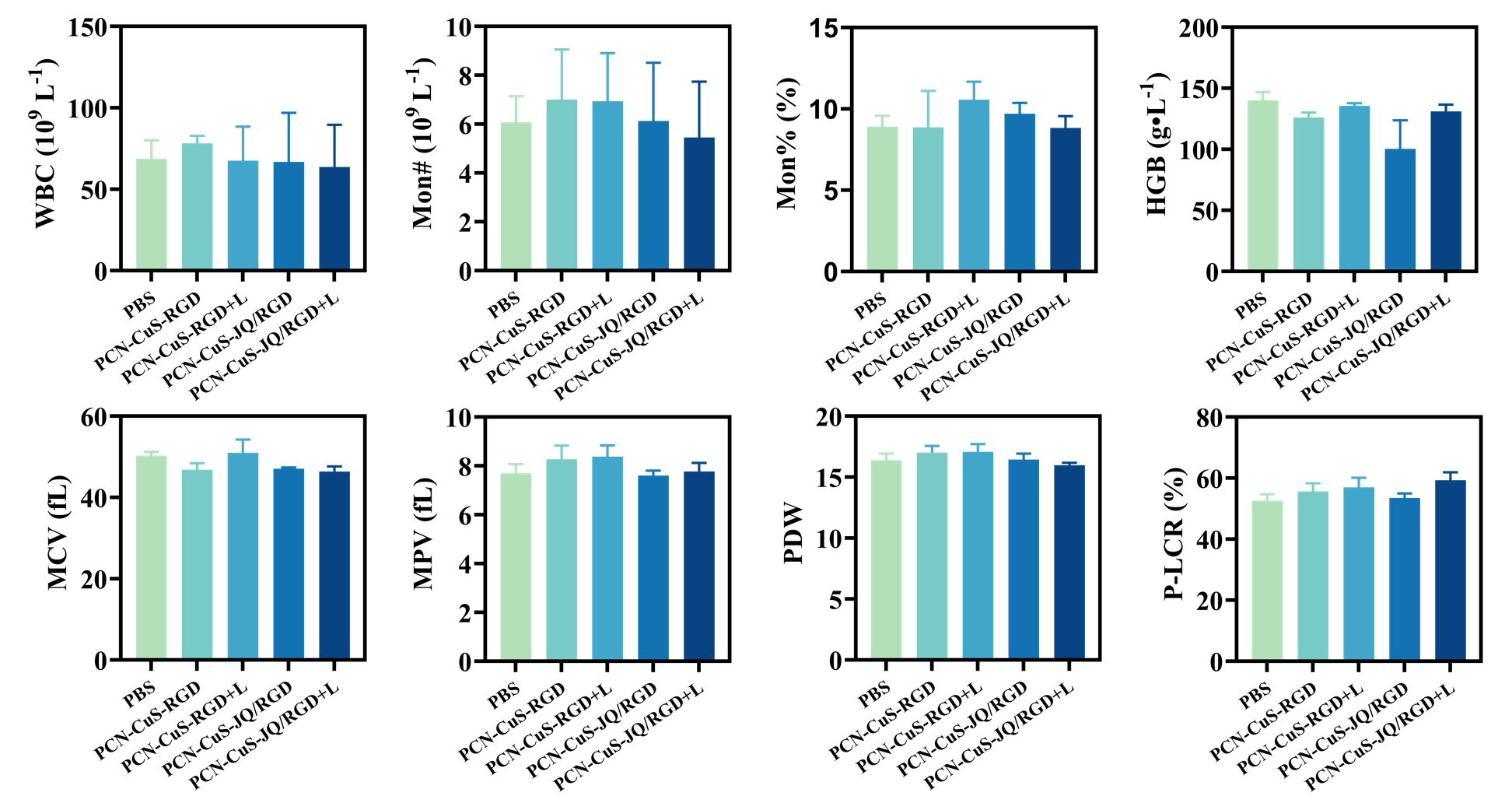


**Figure S18.** Blood routine analysis of mice with different treatments. Data are presented as mean ±  s.e.m. (n=3).


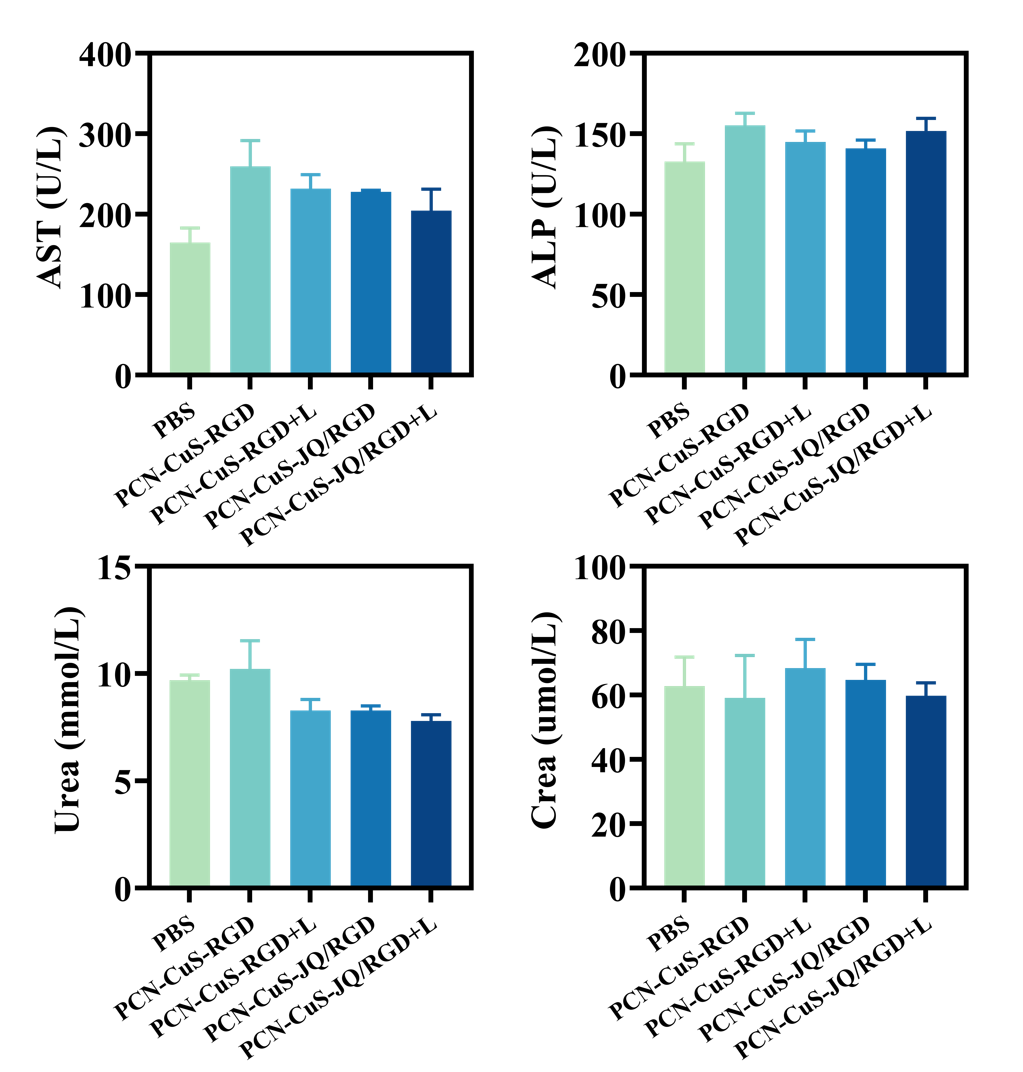


**Figure S19.** Blood biochemistry analysis of mice with different treatments. Data are presented as mean ±  s.e.m. (n=3).


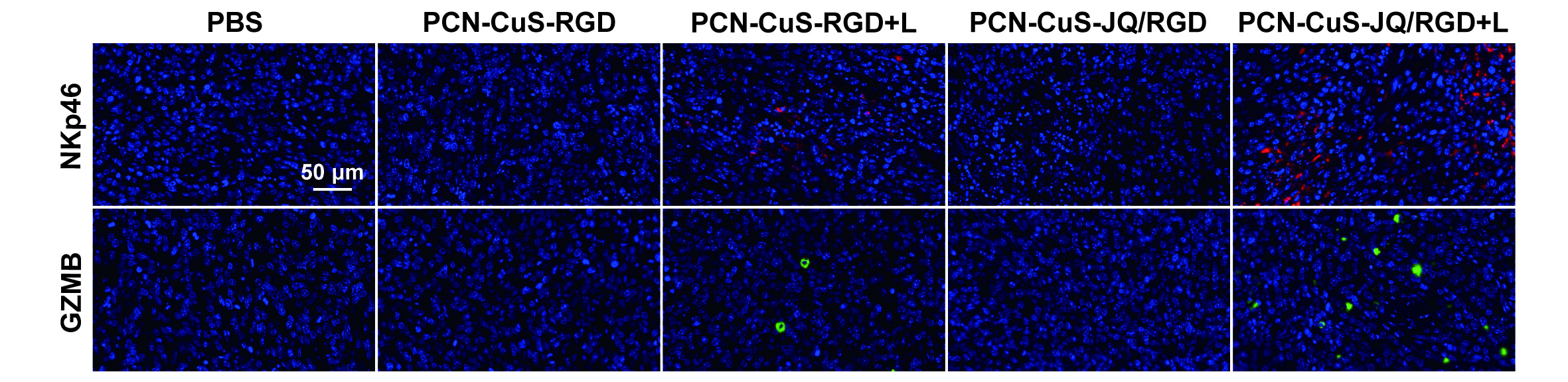


**Figure S20.** Immunofluorescence analysis of NK cell infiltration (NKp46) and cytotoxic effector expression (Granzyme B) in tumor tissues after different treatments.
